# Supplements and Drugs Are Associated With Biological Age in a Cohort of Exceptionally Healthy Individuals

**DOI:** 10.1111/acel.70517

**Published:** 2026-05-21

**Authors:** Kamil Pabis, Weilan Wang, Kumar Selvarajoo, Yelena V. Budovskaya, Vincenzo Sorrentino, Jan Gruber, Brian K. Kennedy

**Affiliations:** ^1^ Healthy Longevity Translational Research Programme, Yong Loo Lin School of Medicine National University of Singapore Singapore City Singapore; ^2^ Department of Biochemistry, Yong Loo Lin School of Medicine National University of Singapore Singapore City Singapore; ^3^ NUS Academy for Healthy Longevity, Yong Loo Lin School of Medicine National University of Singapore Singapore City Singapore; ^4^ Bioinformatics Institute (BII), Agency for Science Technology and Research (A*STAR) Singapore City Singapore; ^5^ Synthetic Biology for Clinical and Technological Innovation (SynCTI) National University of Singapore (NUS) Singapore City Singapore; ^6^ School of Biological Sciences Nanyang Technological University (NTU) Singapore City Singapore; ^7^ TruMe Inc. Alameda California USA; ^8^ Department of Physiology, Yong Loo Lin School of Medicine National University of Singapore Singapore City Singapore

**Keywords:** aging, AKG, biological clocks, coenzyme Q10, epigenetic clock, geroprotectors, polypharmacy, real‐world cohort, supplements

## Abstract

In this cross‐sectional cohort we analyzed data from 4260 “health enthusiasts” who purchased at least one saliva‐based DNA epigenetic test between 2020 and 2025 and completed detailed lifestyle and supplement questionnaires. A proprietary 9‐CpG clock with a mean absolute error of 5.4 years served as the primary biomarker of biological age. High prevalence (71%) of supplement use in this cohort increased our power to study the effects of supplements compared to earlier studies that focused on the general population. We tested the association between 84 commonly used supplements and biological age measured as Age Residual. In our cross‐sectional analysis, a commercially available, delayed‐release calcium‐alpha‐ketoglutarate (dAKG) + vitamin supplement (“Rejuvant”) was associated with an average 1.8‐year lower Age Residual. The difference remained significant in models adjusted for age, sex, smoking, health status and additional covariates. In contrast, participants who reported taking regular AKG showed a much smaller and statistically insignificant benefit. Among medications, there was a non‐significant benefit of antihistamine use, although the analysis was sample‐size limited. In a longitudinal subset, intake of coenzyme Q10 (CoQ10) and dAKG was associated with increased odds of a lower Age Residual, but the results were not significant after multivariate correction. In conclusion, this study underscores the utility of an inexpensive saliva‐based epigenetic test for population‐level aging research and the benefits of health enthusiast cohorts. It highlights AKG and CoQ10, among others, as promising supplements warranting further investigation. Limitations like healthy user and recruitment bias remain and will require future controlled trials to fully address.

## Graphical abstract

Supplement users have lower epigenetic biological age than non‐users. Several supplements or supplement classes are associated with some benefit on epigenetic age in either cross‐sectional or longitudinal analyses.

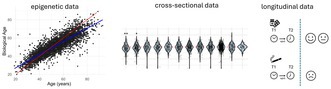

**Abbreviations**
AKG: alpha‐ketoglutarate
ANOVA: analysis of variance
BMI: body mass index
CoQ10: coenzyme Q10
dAKG: delayed‐release alpha‐ketoglutarate
ICC: intraclass correlation coefficient
LLM: large language model
NAD^+^
: nicotinamide adenine dinucleotide
NAD^+^booster: compound or supplement intended to increase cellular NAD levels
NHANES: National Health and Nutrition Examination Survey
NMN: nicotinamide mononucleotide
NR: nicotinamide riboside
OR: odds ratio

## 1 Introduction

Population aging will lead to a strain on healthcare and pension systems worldwide. Treatments to delay aging, extend human lifespan, and improve healthspan are therefore urgently needed to mitigate these economic burdens (Olshansky 2016). Several compounds exist that extend the lifespan in model organisms, including FDA‐approved drugs, phytochemicals, and supplements. However, testing all these compounds in human trials has been slow due to the high costs associated with such studies. Observational studies offer a more affordable way to identify candidate interventions, like drugs or supplements, that could slow aging or delay age‐related morbidity and can be tested in large‐scale controlled trials (Morin et al. 2024; Tang et al. 2023). Data from these studies should be considered as a complement to expensive clinical studies, as they can highlight real‐world effects and discriminate between compounds that should be tested in more controlled settings.

Most earlier studies focused on the intake of supplements like antioxidants (Mursu et al. 2011), multivitamins (Watkins et al. 2000), or minerals (Xiao et al. 2013). More recently, focus has shifted to other commonly used supplements, several of which are known to extend lifespan in preclinical models. For example, glucosamine was found to reduce mortality in multiple observational studies (Bell et al. 2012; Ma et al. 2019; Li, Gao, et al. 2020; Li, Zhong, et al. 2020) and to extend mouse lifespan (Weimer et al. 2014). Similarly, fish oil has been linked with lower cardiovascular and all‐cause mortality in the UK Biobank (Li, Gao, et al. 2020; Li, Zhong, et al. 2020), and decelerated demographic aging in short‐lived NZB/W mice (de Magalhães et al. 2016). Ginseng was associated with (1) lower mortality in the Shanghai Women's Health Study (Pradhan et al. 2022), and (2) lifespan extension in the nematode model *C. elegans* (Wang et al. 2022), although it had no effect on lifespan in mice (Bittles et al. 1979).

Although medication data is routinely collected in modern surveys and electronic medical records, the impact of medications on age‐related outcomes has only recently been studied in observational studies. Cardiovascular, cardiometabolic, and anti‐inflammatory drugs show promise in particular. Calcium channel blockers are associated with improved functional aging (Lopes De Oliveira et al. 2024) and substantial lifespan extension in nematodes (https://orabiomedical.com/mmcleaderboard/), while metformin, angiotensin‐converting enzyme inhibitors, mTOR inhibitors, aspirin, and other anti‐inflammatory drugs are associated with lower frailty in humans and/or modest lifespan benefits in mice (Pabis et al. 2024; Strong et al. 2022, 2008; Thanapluetiwong et al. 2024). Importantly, large‐scale analyses of the UK Biobank have revealed many further drug classes that are associated with lower mortality (Morin et al. 2024).

While mortality and incidence of age‐related disease remain the gold standard to study the efficacy of drugs and supplements, such data may not always be available. Biological age has emerged as an integrative indicator of an individual's overall functional status that, in contrast to mortality, does not require long‐term follow‐up. It can be quantified using a variety of approaches that rely on computational analysis of multidimensional data. Deviations in biological age compared to chronological age predict future mortality and adverse health outcomes as we showed with the LinAge2 clock (Fong et al. 2024) and as other groups showed using the PhenoAge clock (Levine et al. 2018).

To date, only a few relatively small studies have looked at the effects of medications on biological age and even fewer studies investigated supplements. Tang et al. (2023) found that antihypertensives and calcium channel blockers were associated with decreased epigenetic age using different clocks, as did Kho et al. (2021), while another study suggested that antihypertensives can increase epigenetic age (Gao et al. 2018). Among supplements, vitamin D has been linked with lower epigenetic age in both observational and controlled trials (Vetter et al. 2022; Bischoff‐Ferrari et al. 2025), while delayed‐release alpha‐ketoglutarate (AKG), together with vitamin A in males and vitamin D3 in females, branded as Rejuvant, has been shown to reduce epigenetic age in a small human study (Demidenko et al. 2021).

Finally, it is important to point out that few observational studies specifically recruit very healthy participants. Those that did yielded important insights into health‐promoting behaviors and the limits of healthy lifespan. Examples include studies of Seventh‐day Adventists (Orlich et al. 2013), the Nurses' Health Study, and the Physicians' Health Study. We believe that such studies of healthy participants are more likely to uncover *bona fide* aging mechanisms rather than just risk factors for premature mortality.

To bridge knowledge gaps regarding real‐world supplement use and biological age measures, we analyze here a cohort of health enthusiasts that have taken at least one saliva‐based epigenetic clock test and provided data regarding supplement, medication use, and relevant covariates.

## 2 Methods

### 2.1 Recruitment

This cohort study is based on a convenience sample mainly recruited online between 2020 and 2025. All participants had taken at least one TruMe saliva epigenetic age test (TruAge) and data was centrally stored at TruMe. Participants agreed to share their de‐identified data for research purposes and approval for the study was granted under the protocol number NUS‐IRB‐2025‐591. Participants were either referred by supplement and telehealth companies or they directly ordered tests via TruMe. Referral may have included the provision of test vouchers for some, but not all participants.

### 2.2 Data Cleaning

The full cohort consists of 4260 unique participants who provided a valid date of birth and an epigenetic test. Subjects with missing covariates were excluded from the multivariate analysis with a few exceptions. We corrected spelling mistakes, simplified categories, and pooled missing values and undisclosed values into one category, in particular for ethnicity and country of residence. Participants listed their supplements in free‐text form, and these entries were later cleaned up manually and using large language model (LLM)‐assistance (ChatGPT o3). Participants with no entry under supplements are treated as non‐users. For participants with multiple epigenetic tests, we kept the first measurement for the cross‐sectional analysis.

### 2.3 Curation of Supplement Lists and Supplement Counting

Using prior knowledge, visual inspection of the data and LLMs we curated a list of 84 commonly used supplements, supplement classes, medications, and their synonyms. We then performed fuzzy matching to count supplements used by each participant. We included common misspellings and word roots to identify supplements with higher sensitivity (Table S1). Different methods of data cleaning and counting supplements produced comparable results (Figure S2).

In the case of delayed‐release AKG (dAKG), we distinguish between self‐declared dAKG users (questionnaire data), dAKG subscribers (based on order history), and potential dAKG users (referred by PDL Health). All other supplement use was defined based on questionnaires. A simplified recruitment flowchart is shown in Figure S1.

### 2.4 Clock Construction

The TruAge epigenetic clock was constructed by analyzing DNA methylation (DNAm) profiles from saliva samples, focusing on specific loci in the human genome (Demidenko et al. 2021). The clock was designed to be adaptable to self‐sampling, cost‐effective, and provide accurate predictions with a mean absolute error of ≤ 5 years. Saliva was chosen due to its non‐invasive collection method and similar methylation patterns to blood (Zarandooz and Raffington 2025). Methylation quantification was performed using Sanger sequencing or pyrosequencing, both standard high‐resolution DNAm detection methods.

Initially, over 900 age‐related CpG sites were identified from publicly available datasets, which were then narrowed down to a few loci with high predictive capability for donor age. Candidate markers were filtered based on (i) above‐median variation in beta values and (ii) strong correlation between methylation status and chronological age (*R* ^2^ ranging from 0.38 to 0.95). From this pool, nine CpG sites were selected for locus‐specific validation based on their high predictive value and genomic localization within regulatory regions of aging‐associated genes. These sites exhibited methylation changes up to 40% across the human lifespan.

The TruAge clock estimates biological age via a regression‐based formula:

$$\text{Biological}\;\mathrm{Age}=C+\sum_{i=1}^{n}w_{i}\cdot M_{i}$$

where

- *C* is a model intercept (constant),
- *w*_1_…*w*_*n*_ are site‐specific weights (regression coefficients),
- *M*_1_…*M*_*n*_ are the beta values of DNA methylation at each CpG site, and
- *n* is the number of CpG sites used in the model.

The clock was initially developed on a sample of 105 healthy individuals, showing a mean absolute error of 4.67 years, and further refined down to 3.80 years after analyzing data from ~1000 customers. Additionally, a comparison of clock results with those from MyDNAage (ZymoGen) showed a high correlation (*R* = 0.97), demonstrating the reliability and consistency of this saliva‐based clock. Further validation on the current cohort is shown in the results section.

### 2.5 Biological Age Measures and Statistical Analysis

All analyses were performed using R 4.2.2. As biological age measures, we used both Age Delta (Biological Age‐Chronological Age) and Age Residual (residual from a linear regression between Chronological Age and Biological Age), which are less affected by differences in age between treatment groups.

In our univariate comparison of multiple supplements and medications, we corrected for multiple testing using the Benjamini–Hochberg method. Supplements that pass this initial test are then included in a multivariate model. For model 1, we included participants who provided complete data for sex, smoking, and general health (*N* = 3790). For model 2, we only kept participants who provided complete data for the above covariates plus weight, height, physical activity, alcohol consumption, mental health, sleep quality, and stress levels (*N* = 2858). In addition, we corrected for ethnicity, recruiting company, and country of residence in model 2.

Linear model assumptions were tested using the gvlma package (v1.0.0.3) and outliers were excluded afterwards. Matching was performed using the matchit package (v4.7.2), using a nearest neighbor approach with no replacement. Odds ratios for improvement in Age Residual over time were calculated using the epitools package (v0.5.10.1) with the Wald method.

## 3 Results

### 3.1 Baseline Characteristics and Clock Performance

We present here a real‐world cohort of 4260 health enthusiasts who took at least one epigenetic biological age test. We report the baseline characteristics for 3628 of these participants who provided complete questionnaire data in Table 1.

**TABLE 1:** Baseline characteristics.

	This cohort	Fraction	NHANES	Fraction
Timeframe	2020–2025	NA	2017–2018	NA
*N*	3628^a^	NA	5667	NA
Age (mean)	53.5 ± 13.2	NA	50.7 ± 18.3	NA
Biological Age (mean)	51.4 ± 11.1	NA	NA	NA
Smoking				
Yes	136	0.04	1011	0.18
No	3492	0.96	4656	0.82
Health status				
Excellent	1354	0.37	2123	0.37
Good	1827	0.50	2099	0.37
Fair	410	0.11	1195	0.21
Poor	37	0.01	243	0.04
Sex				
Female	1477	0.41	2748	0.48
Male	2151	0.59	2919	0.51
Supplement use^b^				
Yes	2583	0.71	NA	NA
No/no data	1045	0.29	NA	NA

*Note:* Comparison with NHANES 2017 data from our earlier work (Fong et al. 2024). Health status “excellent” in NHANES is the combination of “excellent” and “very good” since our cohort lacks the “very good” category.

^a^ Only participants without missing values were kept.

^b^ Using a broad definition of supplement use.

Compared with the general population, these participants are unusually healthy. For example, the smoking rate is 4% in our cohort as compared to 18% in the National Health and Nutrition Examination Survey (NHANES) and only 12% of our participants report poor or fair health compared to 25% in NHANES, despite somewhat higher mean age in our cohort. Most importantly, our cohort is particularly enriched for supplement users (71%), allowing us to study the correlations between the consumption of rarely used supplements and biological age. Rarely used supplements here are defined as all supplements excluding those only containing vitamins, minerals, omega‐3 fatty acids, and excluding joint supplements, which have been commonly studied in the past. Such rare supplements make up half of all supplements used in this cohort (Figure S3), which is substantially higher than reported in the NHANES and other survey data (Hua et al. 2023; Clarke et al. 2015).

**FIGURE 1:**
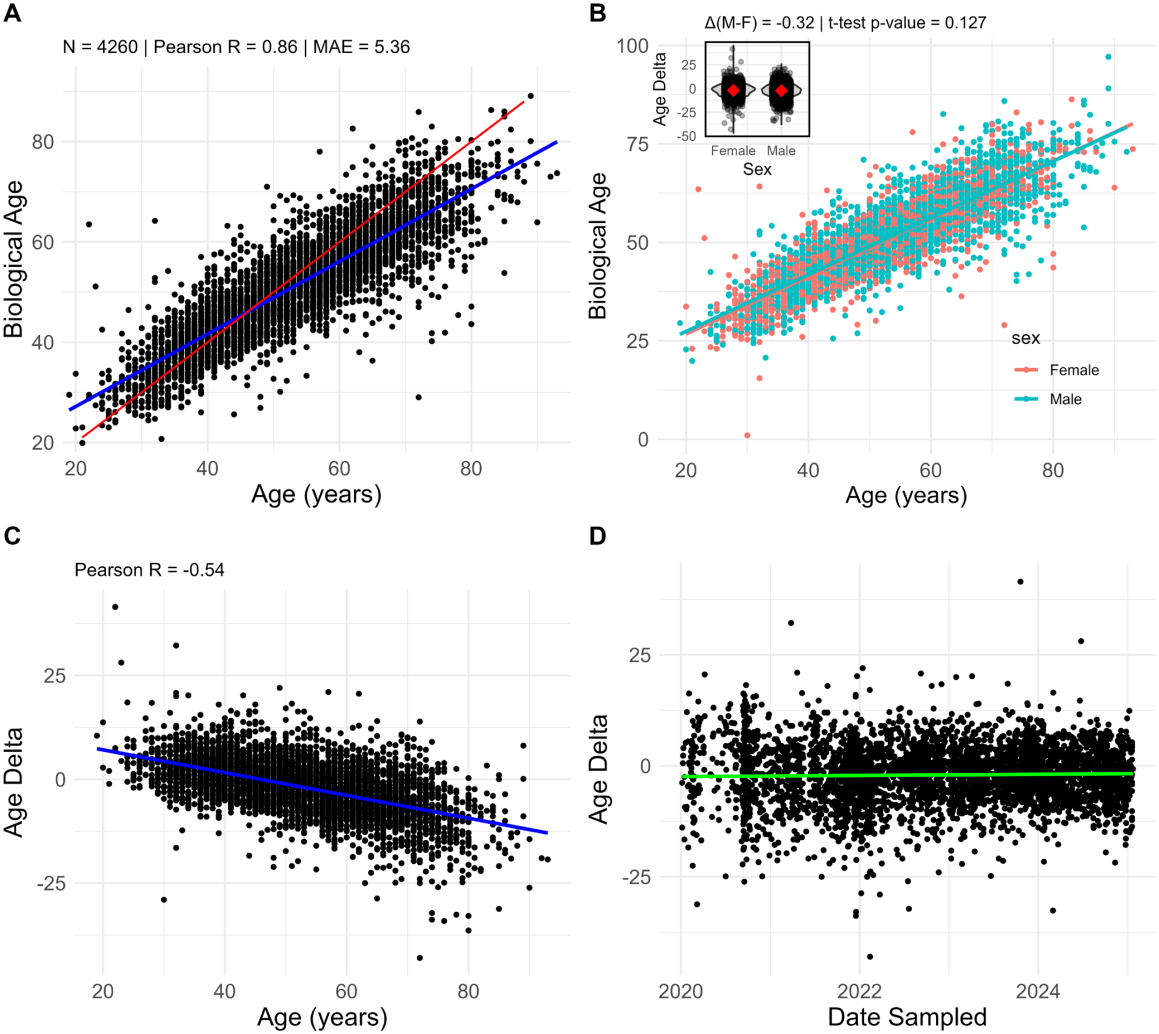
Performance of a proprietary, saliva‐based epigenetic clock. (A) Regression line between Age and Biological Age (*n* = 4260 for all panels) shown in blue and line of *x* = *y* shown in red. (B) Regression line between Age and Biological Age for male (blue) and female (red) participants. Inset shows that the Age Delta (Biological Age—Chronological Age) between males and females is comparable in this cohort (*p* > 0.05 for difference). (C) Regression line between Age and Age Delta (Biological Age—Chronological Age) reveals that older participants are judged to be younger by the saliva‐based epigenetic clock. (D) Scatterplot of tests taken between 2020 and 2025 reveals that the reported Age Delta is stable across time.

The saliva‐based epigenetic biological age in this paper shows a robust association with chronological age, with a mean absolute error of 5.36 years and an *R*‐value of 0.86 across 4260 participants (Figure 1A). No significant differences in biological age and aging trajectories were observed between males and females (Figure 1B). We do, however, note that older subjects are consistently judged to be younger than their chronological age. When we plot the Age Delta, a negative correlation with age is observed (Figure 1C), which can be an artifact of clock construction or true survivor bias. Therefore, in this manuscript we report either Age Delta with adjustment for chronological age whenever possible or Age Residual, which is less sensitive to chronological age (Figure S4). Finally, we show that the reported Age Delta, determined by saliva, is stable over time and not subject to any unexplained drift (Figure 1D).

Although participants in our cohort were recruited via multiple supplement companies and clinics, we do not find any substantial differences in the Age Residual between different recruiters (*p*_ANOVA > 0.05, Figure S5). Similarly, there were no significant differences in Age Residual between ethnicities in this predominantly white/Caucasian cohort (Figure S6). Nationality, in contrast, was significantly associated with Age Residual (*p*_ANOVA < 0.01, Figure S7). Participants from Singapore and Switzerland showed the lowest Age Residual, while participants from Austria had a higher Age Residual.

### 3.2 Multiple Health‐Related Behaviors Correlate With Biological Age

**FIGURE 2:**
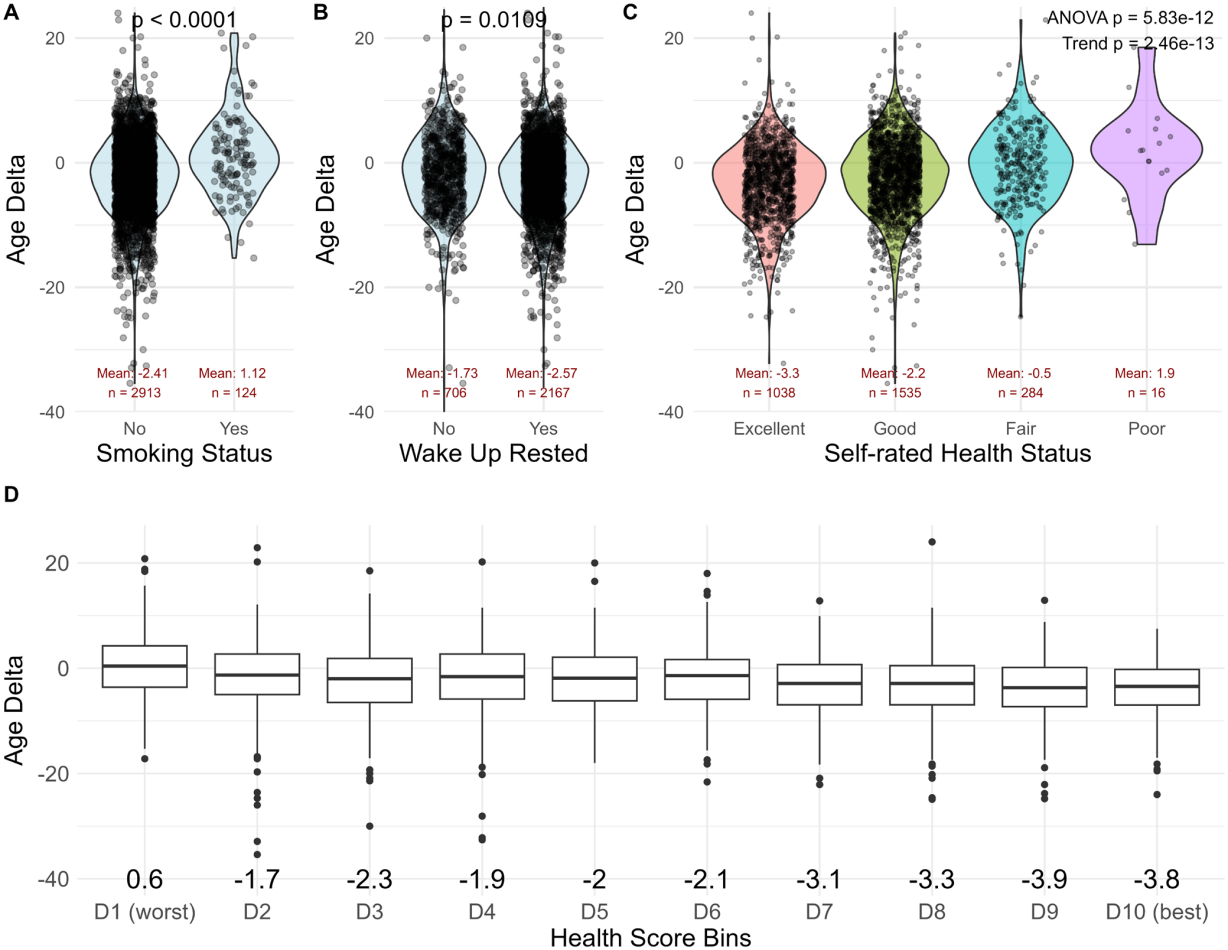
Traditional risk factors are associated with biological age. (A) Smoking is associated with significantly higher Age Delta. (B) Restful sleep is associated with significantly lower Age Delta. (C) Better health status is associated with significantly lower Age Delta. (D) Those in the lowest decile of our health score show a 4.4 year higher Age Delta compared to those in the highest decile. The score includes health status, general mental health status, rested on waking, weekly exercise frequency, smoking status, supplement use, drug use, alcohol intake, and perceived stress level.

In order to gain a better understanding of how this clock behaves in a real‐world cohort, we tested whether traditional risk factors and health behaviors are associated with measures of biological age. As expected, we found that smoking (Figure 2A, Δ3.53 years), low sleep quality (Figure 2B, Δ0.84 years), and low self‐rated health (Figure 2C, Δ5.20 years) were significantly correlated with higher Age Delta. Since multiple factors together may have additive or diminishing effects, we constructed a composite health score based on health status, smoking, mental health status, sleep, exercise, alcohol consumption, stress levels, supplement and medication consumption (Table S2). We found that participants in the most optimal 10th percentile had up to 4.4 years younger Age Delta compared to participants in the bottom 10th percentile (Figure 2D).

The results were similar when using Age Residual (Figure S8), albeit substantially attenuated, suggesting that chronological age mediates some of the associations between health behaviors and Age Delta. Indeed, we find that smoking, health status, sex, country, sleep quality, diabetes, mental health status, body mass index, weekly exercise, stress levels, and alcohol consumption together only explain 2%–3% of the variability in Age Residual (Table S3).

In the following sections, we explore the usage of medications and supplements as unique health behaviors that might impact biological age.

### 3.3 Medication Use and Biological Age in the Cross‐Sectional Cohort

In total, there were 456 participants listing 761 exposures across 22 common medication classes, suggesting our analysis has to be approached with caution due to limited power. The definitions for the medication classes we included are provided in Table S4. It was not known whether medications were prescribed off‐label or on‐label. Nevertheless, consistent with general prescription patterns, we found that metformin users were more likely to be diabetic (Figure S9), users of antidepressant medications were more likely to have lower mental health scores (Figure S10A), and were less likely to wake up rested (Figure S10B).

**FIGURE 3:**
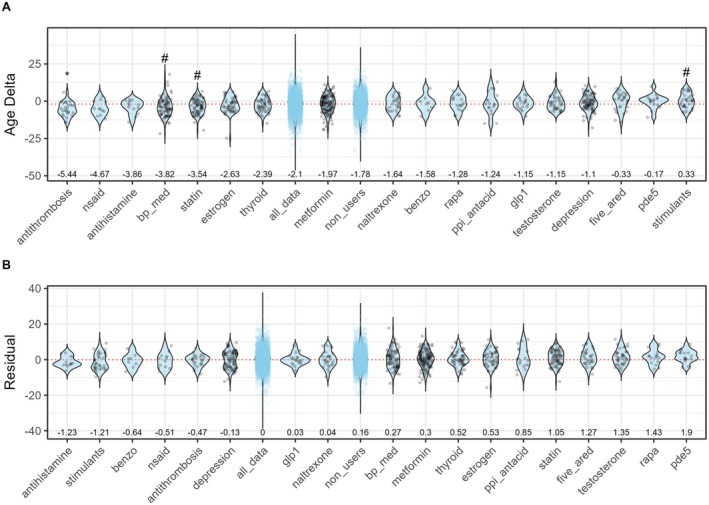
Association between medication classes and measures of biological age. The impact of different medication classes on Age Delta is shown in (A) and the impact of different medication classes on Age Residual in (B). The median of the control group (all_data) is indicated with a dashed red line. Control group and medication non‐users (non_users) are plotted for comparison in light blue. * and # indicates groups with a *p* < 0.05 or *p* < 0.10, respectively, after Benjamini–Hochberg correction when compared with all participants. Mean biological age shown on top of the *x* axis for each group, *N* = 8–51 per group. antithrombosis, antithrombotic agents; benzo, benzodiazepines; bp_med, antihypertensive agents; depression, antidepressants; five_ared, 5α‐reductase inhibitors; glp1, glucagon‐like peptide‐1 receptor agonists; naltrexone, opioid receptor antagonists; nsaid, non‐steroidal anti‐inflammatory drugs; pde5, phosphodiesterase‐5 inhibitors; ppi_antacid, proton‐pump inhibitors/antacids; rapa, rapamycin; stimulants, central nervous system stimulants; thyroid, thyroid hormone replacement therapy.

Despite sample size limitations, we found several suggestive relationships. In our initial analysis, statins (*p* < 0.10), antithrombosis (*p* < 0.05), and blood pressure medications (*p* < 0.10) were associated with lower Age Delta after correction for multiple testing, while central nervous system stimulants were associated with higher Age Delta (*p* < 0.10, Figure 3A). No clear benefit or harm was observed for rapamycin, metformin, or GLP‐1 agonists, although these have been linked with improved health outcomes in mouse and human studies before. The broader antithrombosis category included drugs such as aspirin, warfarin, or clopidogrel, while antihypertensives included diuretics, angiotensin‐converting enzyme (ACE) inhibitors, angiotensin II receptor blockers, and other commonly prescribed classes. Biological age for individual drugs instead of classes is shown in Figure S11A,B.

However, it is important to consider that drug prescribing patterns can vary across age groups. When we correct for age and basic covariates (sex, smoking, general health) most of the results are attenuated. This is also evident when we plot the Age Residuals instead of the Age Delta because the residuals are more stable with participant age (Figure 3B).

### 3.4 Supplement Use and Biological Age

**FIGURE 4:**
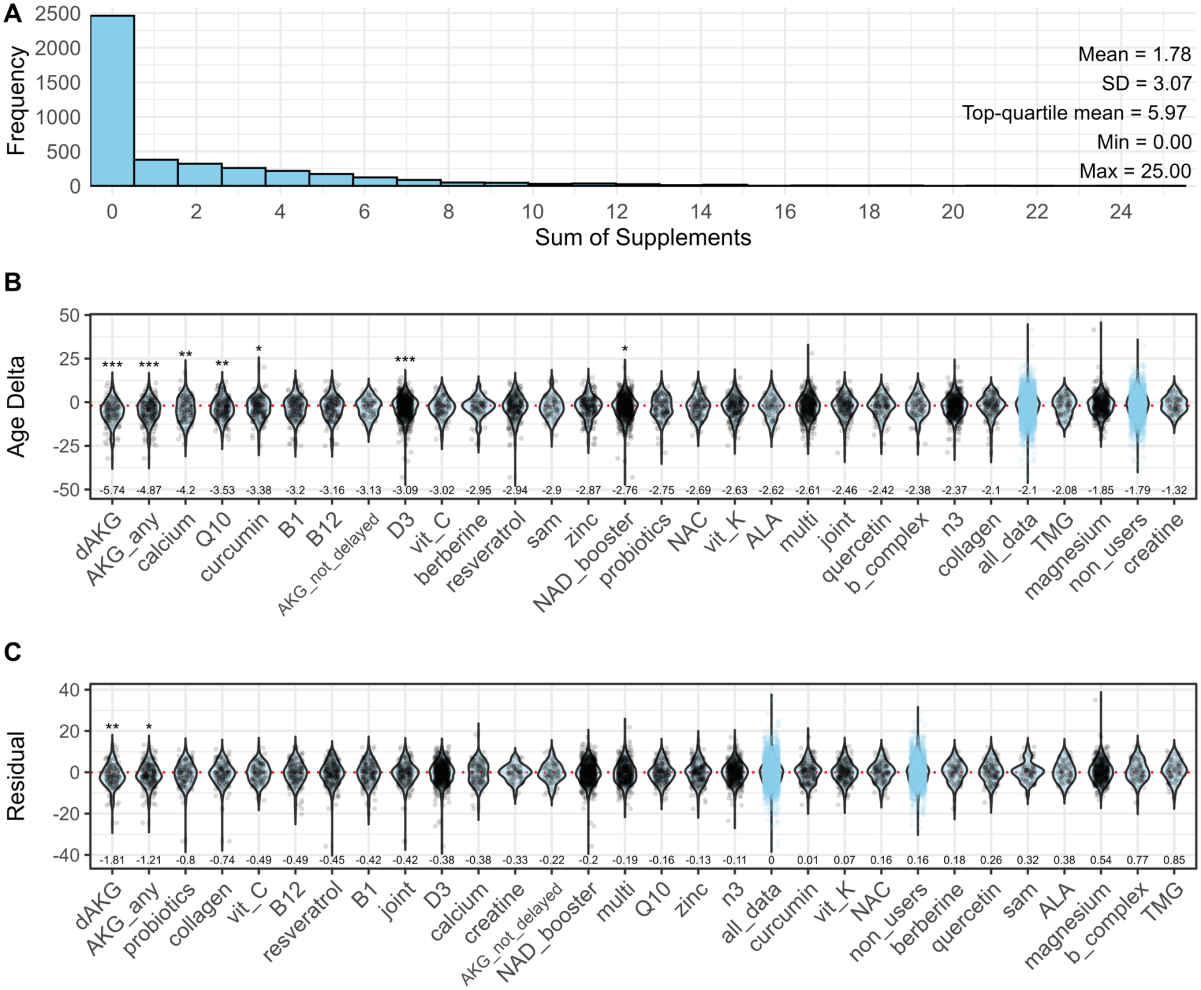
Impact of supplements on biological age. Most participants consume between zero and three supplements with a long tail of high consumers (A). The impact of supplements or supplement classes on Age Delta is shown in (B) and on Age Residual in (C) where lower residual means younger than expected. The median of the control group (all_data) is indicated with a dashed red line. Control group (all_data)_and all supplement non‐users (non_users) are plotted for comparison in light blue. Groups with *N* > 70 users shown. *, **, *** indicates groups with a *p* < 0.05, *p* < 0.01 or *p* < 0.001 after Benjamini–Hochberg correction when compared with all participants. Mean biological age shown on top of the *x* axis for each group. AKG_any, α‐ketoglutarate (any form); AKG_not_delayed, α‐ketoglutarate (non‐dAKG); ALA, α‐lipoic acid; b_complex, full B‐complex; B1, vitamin B1; B12, vitamin B12; D3, vitamin D; dAKG, branded calcium α‐ketoglutarate (Ca‐AKG); joint, joint‐support blend (glucosamine, chondroitin, MSM, etc.); multi, multivitamin/mineral blend; n3, omega‐3 fatty acids; NAC, N‐acetyl‐L‐cysteine; NAD_booster, NAD^+^ precursors (e.g., NMN or NR); Q10, coenzyme Q10; sam, S‐adenosyl methionine; TMG, trimethylglycine/betaine; vit_C, vitamin C; vit_K, vitamin K. *N* = 4260 for panels (B) and (C).

In total there were 1762 supplement users listing 7662 supplements from our list of common supplements (1.8 ± 3.1 supplements taken per person and 4.2 ± 3.5 per supplement user, Figure 4A, Figure S12). The top 5 most common supplements consumed in this cohort were vitamin D, various “nicotinamide adenine dinucleotide (NAD^+^) boosters”, omega‐3 fatty acids, magnesium, and multivitamins. A table with the full list is provided for all supplements taken by more than 30 people (Table S5).

During this validation step, we surprisingly found that supplement users showed a significantly younger Age Residual (Figure S13A) and Age Delta (Figure S13B) than non‐users, while this was not the case for medication users (Figure S13C,D). Heavy supplement use was neither beneficial nor harmful compared to moderate supplement use (Figure S14A,B).

We found that alpha‐ketoglutarate (AKG), carotenoids, calcium, coenzyme Q10 (CoQ10), curcumin, vitamin D3, and NAD^+^ booster supplements were associated with lower Age Delta after correction for multiple testing (Figure 4B). Our analysis also distinguishes between AKG and a proprietary, delayed‐release AKG (dAKG) sold under the brand name “Rejuvant” that contains additional vitamins. We compare any AKG intake (“AKG_any”), which includes dAKG and other AKG formulations, and non‐dAKG intake (“AKG_not_delayed”). Intriguingly, lower Age Delta was only seen in the two groups including dAKG. While AKG_not_delayed showed a trend toward lower Age Delta, the difference did not reach significance.

Supplement users are different from non‐users in their distribution of covariates (Table S6). Especially for dAKG, we find that users were older than non‐users (Table S7), which could bias the Age Delta. When we correct for the impact of this chronological age effect by using Age Residual, none of the supplements maintain an association with biological age except for dAKG and AKG (Figure 4C). Similarly, there were no significant associations for different subtypes of vitamin D, vitamin K, NAD^+^ precursors (nicotinamide mononucleotide [NMN], nicotinamide riboside [NR], niacin), and carotenoids (Figure S15). We did, however, find that Tru Niagen NR consumption was associated with a significantly lower Age Residual before correction for multiple testing, consistent with a nominal benefit of NR (data not shown).

Next, we corrected for multiple confounders using a matching approach or a linear model. The covariates included were age, smoking, health status, sex, country, recruiting brand, sleep quality, mental health, BMI, exercise, stress level, and alcohol consumption (Figure S16A). Using the matching approach, dAKG users had a 1.27 years lower Age Residual compared to matched non‐users (*p* < 0.01, Figure S16B). When we corrected for these covariates using a linear model, dAKG also remained associated with Age Residual (*p* < 0.01, *n* = 2480 participants, adjusted *R* ^2^ = 0.009, Table S8).

Since we know that some of the participants were on a dAKG subscription plan, even though they may not have disclosed the supplement in the questionnaire, we repeated our analysis focusing on this subgroup. Surprisingly, we found that dAKG subscribers had an Age Residual that was comparable to non‐users (Figure S17).

### 3.5 Impact of Delayed‐Release Alpha‐Ketoglutarate Supplement on Different Subgroups

We performed an analysis for moderators of dAKG effects on Age Residual, but none of the results reached significance. Since these results are likely sample size limited, we provide additional descriptive data for subgroups below, including sex, health score, weight, chronological age, smoking, and exercise.

**FIGURE 5:**
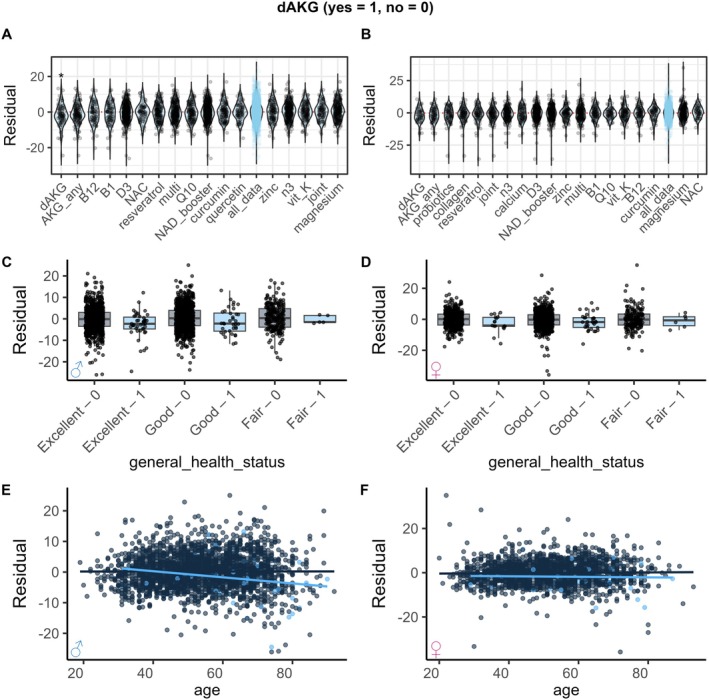
dAKG is associated with lower biological age across different subgroups. (A, B) dAKG is the supplement associated with the lowest Age Residual in both men (A, *n* = 2242, *p*_unadj = 0.0027, *p*_adj = 0.049) and women (B, *n* = 1762, *p*_unadj = 0.020, *p*_adj = 0.30). Groups with *N* > 75 users shown for men and *N* > 50 users shown for women. (C, D) dAKG (in blue) is associated with lower Age Residual in men (C, *n* = 2148) and women (D, *n* = 1467) across different levels of health status. There were not enough participants in the “poor” health category for a comparison. (E, F) dAKG (in blue) is associated with lower Age Residual in men (E, *n* = 2489) and women (F, *n* = 1762), although it appears that older men benefit more from dAKG. AKG_any, α‐ketoglutarate (any form); B1, vitamin B1; B12, vitamin B12; D3, vitamin D; dAKG, branded calcium α‐ketoglutarate (Ca‐AKG); joint, joint‐support blend (glucosamine, chondroitin, MSM, etc.); multi, multivitamin/mineral blend; n3, omega‐3 fatty acids; NAC, N‐acetyl‐L‐cysteine; NAD_booster, NAD^+^ precursors (e.g., NMN or NR); Q10, coenzyme Q10; vit_K, vitamin K.

In total there were 143 dAKG users in our cohort based on the questionnaire data. dAKG was the supplement associated with the lowest Age Residual in both men (Figure 5A) and women (Figure 5B). When adjusting for basic covariates, both male (Figure S18A) and female dAKG users (Figure S18B) had significantly lower Age Residual. dAKG was also beneficial across health scores (Figure 5C,D), and across age groups (Figure 5E,F). However, older men appeared to respond better than older women. There were not enough smokers for a subgroup analysis.

Additionally, we found that dAKG was associated with lower Age Residual regardless of weight (Figure S19A), weekly exercise volume (Figure S19B) or alcohol intake (Figure S19C), with slightly larger benefits seen in those who exercise more often. In the subset of participants recruited via either TruMe Labs (Figure S20A) or PDL Health (Figure S20B), dAKG and AKG each ranked among the top supplements, indicating the results were not driven by a single sub‐cohort.

### 3.6 Analysis of Longitudinal Trajectories in Biological Age

A subset of participants took multiple tests, allowing us to evaluate longitudinal trajectories in biological Age Residual (*n* = 755). Given the small sample size, the results should be interpreted with caution.

In principle, multiple measurements would allow us to assess the technical reliability of the test, e.g., intraclass correlation coefficient (ICC). Since very few participants took a test on the same day, however, it is difficult to determine the within‐day ICC. Instead we can estimate the ICC for tests taken a few weeks apart, which is between 0.45 and 0.5 and declines for tests at longer intervals (Figure S21). This value is lower than for blood‐based epigenetic clocks (Belsky et al. 2022) but not dissimilar to the ICC for blood pressure (Palatini et al. 2025; Yong et al. 2017), which is a reliable tool for population‐level research.

The mean time between tests was 367 days (range 0–1741). Over time, 472 participants showed a reduction in Age Residual while only 283 showed an increase (a representative subset is shown in Figure S22). Although most supplements (73%) were associated with an improvement in Age Residual, taking any supplement was not associated with a higher probability of reduced Age Residual (odds ratio [OR]: 0.86 [0.64–1.16], *p* = 0.322, *n* = 332). As expected, smoking was associated with a lower probability of improved Age Residual (OR: 0.42 [0.18–0.95], *p* = 0.037, *n* = 24).

**FIGURE 6:**
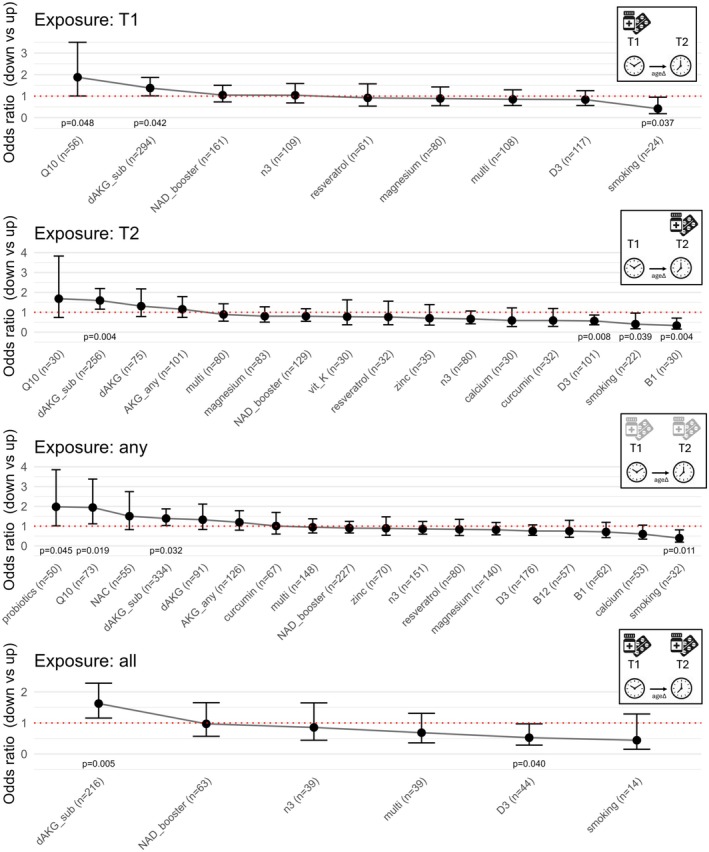
Supplement use and biological age changes over time. CoQ10 and dAKG supplements are associated with a favorable odds ratio of having a decreased Age Residual. This is true when supplement exposure precedes the last measurement of biological age (A; *n* ≥ 50), when it coincides with the last measurement (B; *n* ≥ 30), when it either precedes or coincides (C; *n* ≥ 50), or when supplement exposure both precedes and coincides with the last measurement of biological age (D; *n* ≥ 30). All data are based on self‐reported supplement use. Data for smoking and dAKG subscription is shown for comparison. Odds ratio and *p*‐value were calculated based on the Wald method and not adjusted for multiple comparisons. AKG_any, α‐ketoglutarate (any form); B1, vitamin B1; B12, vitamin B12; D3, vitamin D; dAKG, branded calcium α‐ketoglutarate (Ca‐AKG); dAKG_sub, dAKG subscription; multi, multivitamin/mineral blend; n3, omega‐3 fatty acids; NAC, N‐acetyl‐L‐cysteine; NAD_booster, NAD^+^ precursors (e.g., NMN or NR); Q10, coenzyme Q10; vit_K, vitamin K.

Participants who used CoQ10 (OR: 1.87 [1.01–3.50], *p* = 0.048, *n* = 56) at baseline were almost twice as likely to show an improvement in Age Residual than non‐users (Figure 6A). While dAKG stood out in the cross‐sectional analysis as beneficial, it did not lead to a significant improvement in the longitudinal analysis. This may have been due to the limited sample size with only 26 participants taking dAKG based on the survey data.

Since we know that some of the participants were on a dAKG subscription plan, even though they may not have declared the supplement in the questionnaire, we repeated our analysis focusing on this subgroup. We found that participants on a dAKG subscription were more likely to show improvement in Age Residual (OR: 1.37 [1.01–1.89], *p* = 0.045, *n* = 294). dAKG subscription was the only supplement that was significantly associated with an improvement in Age Residual after we adjusted for age, smoking, sex, general health and length of use, but the result became non‐significant after correcting for baseline Age Residual and regression to the mean (Table S9). Importantly, we found that dAKG subscribers took the same number of epigenetic tests as participants who were referred by other brands (Figure S23A) and at similar intervals (Figure S23B), arguing against healthy user bias.

Finally, we explored whether changing the definition of supplement exposure affects the results. In our initial analysis, we assumed that the supplement exposure at time point T1 must precede the final measurement of biological age at time point T2, but in practice users may have stopped between T1 and T2, while those who only declared supplement use at T2 likely started between T1 and T2. This analysis (Figure 6B–D) generally agrees with our initial approach, finding that CoQ10 and dAKG subscription users are more likely to show improved Age Residual over time.

## 4 Discussion

We show here for the first time that an inexpensive, non‐invasive, saliva‐based biological age test can be a powerful tool to conduct population research and uncover relationships between lifestyle, medications, and supplement use.

The biological age test utilized in this study is a first‐generation epigenetic clock (Johnson and Shokhirev 2025) as it was trained on chronological age. Thus, it should not be surprising that health behaviors have a modest impact on biological age in this study, with age and smoking being the largest influences, since first generation clocks have been shown to be less sensitive to interventions (Sehgal et al. 2024).

A key strength and innovation of this study is our focus on health enthusiasts. Participants in our cohort are unusually healthy, with a biological age reduction of over 2 years relative to their chronological age. Chronologically older participants, in particular, are biologically younger than expected which may be caused by a combination of differential survivorship and statistical effects (Marioni et al. 2019). Such a healthy cohort profile allows us to study potential effects on markers of aging with reduced confounding by disease, and also to study a large variety of supplements, since approximately half of the participants in this cohort take non‐multivitamin, non‐mineral supplements.

Nevertheless, we failed to detect any significant association with biological age for the majority of the 84 supplement classes we studied. However, in the cross‐sectional analysis those who took any supplement showed significantly lower biological age and there was also a non‐significant benefit of taking supplements in the longitudinal analysis. While this could represent healthy user bias, it could also represent a real, albeit small effect. For example, in our re‐analysis of the mouse Interventions Testing Program (Pabis et al. 2024) we find that, even excluding significant results, 60% of the interventions (54 out of 88) increase lifespan, consistent with this hypothesis.

Medication data was available only for a limited subset of participants. With that shortcoming in mind, it is interesting to note that antihistamines were associated with non‐significantly lower Age Delta (rank 3) and Age Residual (rank 1). In other studies, some classes of antihistamines are associated with lower mortality (Morin et al. 2024) and improved cancer survival (Fritz et al. 2021), highlighting potential benefits of this drug class.

Our data contrasts with the polypharmacy literature. Traditionally, doctors view polypharmacy as a liability to be avoided (Chen et al. 2024). However, it is more likely that polypharmacy is a consequence and not a cause of age‐related morbidity. Several observational studies, for example, found that combining two drugs reduces all‐cause mortality (Gray et al. 2011; Tan et al. 2020). The potential risks and harms of supplement polypharmacy are less understood. In our cohort almost 11% of the participants were taking more than 5 supplements, yet their health condition was excellent and higher supplement use was not associated with older biological age, suggesting that supplement combinations are well tolerated.

In contrast to the DO‐HEALTH study, we did not find any significant benefits of omega‐3 fatty acids or vitamin D on aging clocks (Bischoff‐Ferrari et al. 2025; Vetter et al. 2022). However, since the benefits seen in DO‐HEALTH were small and inconsistent among clocks, we may have lacked power to detect true differences. Perhaps in line with this idea, we did see a nominally significant benefit of vitamin D on biological age (*p* = 0.037) before correcting for multiple testing. Similarly, although NAD^+^ metabolism has been implicated in diseases of aging preclinically (Yusri et al. 2025), we did not find significant benefits of various NAD^+^ boosters on biological age, with the exception of branded nicotinamide riboside, which was associated with lower biological age before correcting for multiple testing.

On the other hand, we did find tentative evidence for the benefits of two supplement classes, AKG and CoQ10. AKG is an intermediate in the citric acid cycle, a metabolic precursor for the synthesis of certain amino acids, and a co‐factor in collagen synthesis and epigenetic modifying enzymes (Gyanwali et al. 2022). The studied dAKG supplement is a proprietary delayed‐release version of calcium‐AKG with additional vitamin A and vitamin D3 for men and women, respectively.

We found that dAKG use was associated with lower biological age in our cross‐sectional analysis, while there was no significant benefit in the underpowered longitudinal analysis. However, we did find that participants who were on a dAKG subscription, a much larger group than self‐declared dAKG users, were significantly more likely to show a reduction in biological age over time in our unadjusted analysis. Our results are broadly consistent with reduced biological age reported by Demidenko et al. (2021).

Although we tried to adjust for known confounders, given our study design we cannot exclude healthy user and selection biases. Although our cross‐sectional analysis compares all participants based on their self‐declared supplement use in a survey, among those who were recruited via PDL Health, a large subset received a free epigenetic test upon dAKG purchase, potentially affecting or reflecting health‐seeking behavior. However, the fact that dAKG was also a top supplement that reduced biological age (significant before multiple testing) in the TruMe cohort, which does not suffer from the same recruitment biases, strengthens our confidence in those findings.

Similarly, in the longitudinal analysis, it is possible that participants on a dAKG subscription were more motivated than other participants. We consider this less likely since all the participants included in this analysis had to take at least two epigenetic tests, suggesting a high level of motivation across all participants.

A benefit on biological age is certainly plausible. Plasma AKG declines with aging in mice (Pilley et al. 2024), AKG extends fly lifespan on a low protein diet (Demianchuk et al. 2024) and Ca‐AKG supplementation late in life decreases frailty and extends mouse lifespan (Shahmirzadi et al. 2020), although a later mouse study did not confirm this (Miller et al. 2024), highlighting the need for further research. Currently ongoing mouse studies from the Interventions Testing Program and human studies like the ABLE trial (Sandalova et al. 2023) may help to clarify the role of AKG and/or dAKG in longevity.

CoQ10 is a lipid‐soluble antioxidant and mitochondrial electron carrier. We found that CoQ10 use at baseline was associated with significantly better odds of reduced biological age at follow‐up, even though CoQ10 was not beneficial in the cross‐sectional analysis. This result certainly merits follow‐up given the multifaceted role of CoQ10 in longevity. CoQ10 levels decline with aging in mouse and human tissues (Kalén et al. 1989) and, based on this, multiple groups tested whether CoQ10 supplementation can extend the lifespan of rats and mice, generally with negative results (Sohal et al. 2006; Lönnrot et al. 1998). In humans, on the other hand, CoQ10 has been found to improve cardiometabolic risk factors (Liu et al. 2022) and decrease mortality in heart failure patients (Xu et al. 2024).

## 5 Limitations

There are several limitations of our study design. Our cohort is predominantly Caucasian and we only have limited and heterogeneous data on self‐reported ethnicity. Thus, we do not know how the findings may generalize to other populations. Since the cohort is based on a convenience sample, it is more difficult to exclude recruiting and healthy user bias, leading to residual confounding. The provision of vouchers to some dAKG customers could further contribute to confounding, although we tried to correct for this.

In addition, our longitudinal analysis is small and limited by sample size. Self‐reported supplement use may be biased, and we use LLM assisted fuzzy matching for supplement and medication names. Frequency and dose of supplements were not reported. Finally, the epigenetic clock utilized in this study may underestimate the benefits of supplements since it is a less sensitive first generation‐like clock.

## 6 Conclusion

Our data is consistent with the hypothesis that CoQ10 and dAKG supplementation reduce biological age. We suggest that future studies leverage health enthusiasts and easily accessible biomarkers of aging to identify novel gerotherapeutics with increased study power.

## Author Contributions

K.P.: data analysis, writing. B.K.K.: conception, writing, funding. J.G., V.S., and K.S.: writing, funding. W.W.: writing, data analysis. Y.V.B.: resources and data curation (cohort maintenance).

## Funding

The authors have nothing to report.

## Conflicts of Interest

The authors declare no conflicts of interest.

## Supporting information


**Figure S1:** Subject recruitment flowchart.
Most participants bought a TruMe epigenetic test online. Some participants recruited through PDL Health received a gift voucher for a free test. Please note that not all dAKG supplement users were recruited through PDL Health. Conversely, not all who were recruited via PDL Health necessarily took or declared taking dAKG supplement at the time of the survey.
**Figure S2:** Comparing methods for data cleaning. We show here that two methods of cleaning and counting supplements produce very similar total counts for supplements. In method 1, common typographic mistakes are corrected by hand while in method 2, all mistakes are automatically corrected using ChatGPT o3.
**Figure S3:** Percentage of “rare” and “common” supplements taken by participants. Rare supplements here are defined as all supplements that are not exclusively containing vitamins, minerals, omega‐3 fatty acids and are not joint support supplements. These supplements have been rarely studied in prior studies.
**Figure S4:** Age Residual vs chronological age. The mean Age Residual is stable across different age strata.
**Figure S5:** Age Residual by recruiter (supplement brand or clinic). The Age Residual for participants recruited via different companies, longevity clinics and supplement brands varies moderately and non‐significantly. Showing brands with more than 40 data points. *N* = 3641.
**Figure S6:** Age Residual by ethnicity. The Age Residual for participants across different ethnicities is comparable.
**Figure S7:** Age Residual by country. We observe significant differences in Age Residual across participants from different countries in our cohort.
**Figure S8:** Traditional risk factors are associated with biological age. (A) Smoking is associated with significantly higher Age Residual. (B) Restful sleep is associated with non‐significantly lower Age Residual. (C) Better health status is associated with lower Age Residual. (D) Comparison of participants in the lowest and highest decile of our health score that includes general health status, general mental health status, rested on waking, weekly exercise frequency, smoking status, supplement use, drug use, alcohol intake and perceived stress level.
**Figure S9:** Different medications are associated with diabetes prevalence. Here we show the prevalence of diabetes (“has diabetes” coded as 2) across different medication classes. Corrected for multiple testing using Benjamini–Hochberg. *N* ≥ 15 per group. stimulants = central nervous system stimulants, nsaid = non‐steroidal anti‐inflammatory drugs, ppi_antacid = proton‐pump inhibitors/antacids, naltrexone = opioid receptor antagonists, benzo = benzodiazepines, bp_med = antihypertensive agents, glp1 = glucagon‐like peptide‐1 receptor agonists, depression = antidepressants, antithrombosis = antithrombotic agents, five_ared = 5α‐reductase inhibitors, pde5 = phosphodiesterase‐5 inhibitors, rapa = rapamycin, thyroid = thyroid hormone replacement therapy.
**Figure S10:** Antidepressant use is associated with mental health and sleep quality. (A) Antidepressant use is associated with significantly worse mental health scores. A higher score means worse mental health. (B) Antidepressant use is associated with significantly less restful sleep. 1 = waking up tired, 2 = waking up rested. Data was corrected for multiple testing using Benjamini–Hochberg. *N* ≥ 15 per group. stimulants = central nervous system stimulants, nsaid = non‐steroidal anti‐inflammatory drugs, ppi_antacid = proton‐pump inhibitors/antacids, naltrexone = opioid receptor antagonists, benzo = benzodiazepines, bp_med = antihypertensive agents, glp1 = glucagon‐like peptide‐1 receptor agonists, depression = antidepressants, antithrombosis = antithrombotic agents, five_ared = 5α‐reductase inhibitors, pde5 = phosphodiesterase‐5 inhibitors, rapa = rapamycin, thyroid = thyroid hormone replacement therapy.
**Figure S11:** Association between individual medications and measures of biological age. The impact of different drugs on Age Delta is shown in (A) and the impact of different drugs on Age Residual in (B). The list of drugs includes commonly used drug names and abbreviations. The median of the control group (all_data) is indicated with a dashed red line. Control group and drug non‐users (non_users) are plotted for comparison in light blue. * and # indicate groups with a *p* < 0.05 or *p* < 0.10, respectively, after Benjamini–Hochberg correction when compared with all participants. Mean biological age shown on top of the *x* axis for each group.
**Figure S12:** Distribution of supplement consumption in our cohort. Most participants consume between zero and three supplements with a long tail of high consumers.
**Figure S13:** Impact of supplements and medications on biological age. Supplement users have significantly lower Age Residual (A) and Age Delta (B). In contrast, those who list using medications on the survey do not show reduced biological age measures (A, B). *N* = 3790.
**Figure S14:** Heavy supplement use is not associated with adverse effects on biological age.
Heavy supplement users (5+ different supplements taken) have similar Age Residual (A) but lower Age Delta (B) compared with non‐users and moderate users.
**Figure S15:** Impact of supplements on biological age. Certain supplements are associated with lower biological age. The median of the control group (all_data) is indicated with a dashed red line. Control group and all supplement non‐users (non_users) are plotted for comparison in light blue. *, **, *** indicates groups with a *p* < 0.05, *p* < 0.01 or *p* < 0.001 after Benjamini–Hochberg correction when compared with all participants. Mean biological age shown on top of the *y* axis for each group (lower Age Delta or Residual means younger than expected). lycope = lycopene; NR = nicotinamide riboside; D3 = vitamin D3 (cholecalciferol); NMN = nicotinamide mononucleotide; caroten2 = β‐carotene; vit_k2 = vitamin K2 (menaquinone); astaxa = astaxanthin; niacin = niacin (vitamin B3); D2 = vitamin D2 (ergocalciferol); lutein = lutein; vit_k1 = vitamin K1 (phylloquinone).
**Figure S16:** dAKG users show a lower biological age compared to non‐users. Matching balances covariate distribution across groups (A). dAKG is associated with lower Age Residual even when compared to matched controls (B).
**Figure S17:** dAKG subscribers show a similar biological age to non‐users. Actual dAKG users show the lowest Age Residual while dAKG subscribers are closer to non‐users. Actual dAKG users are those who mention dAKG in the supplement questionnaire, dAKG subscribers have a subscription plan, while possible dAKG users bought dAKG at one point in time. Non‐dAKG users includes all participants that fit neither of those three categories.
**Figure S18:** dAKG effects on biological age in men and women. After adjusting for age, smoking, general health status and brand we find that dAKG is associated with significantly lower Age Residual in men (A) and women (B).
**Figure S19:** The effects of dAKG on biological age in different subgroups. dAKG use (in blue) is associated with decreased Age Residual regardless of weight (A), weekly exercise volume (B) and amount of alcohol consumed (C). Those who exercise regularly might benefit more from dAKG.
**Figure S20:** dAKG is associated with lower biological age in two different sub‐cohorts. Only supplements with more than 40 users are shown in the figure. (A) dAKG supplement lowers Age Residual in the TruMe sub‐cohort (*p*_unadj = 0.036, *p*_adj = 0.53). (B) dAKG supplement significantly lowers Age Residual in the PDL Health sub‐cohort (*p*_unadj = 0.00072, *p*_adj = 0.0096).
**Figure S21:** Test to test variability of the saliva epigenetic clock. The intraclass correlation coefficient (ICC) is calculated against a running cut‐off. We calculate the ICC for all participants that had two epigenetic tests less than X days apart (“Max Days Between Tests” shown on the *X*‐axis). As expected, the higher the time elapsed between tests the lower the correlation between two given tests on average. Fluctuations, especially, at earlier time points may be due to low sample sizes.
**Figure S22:** Biological age trajectories for a random subset of participants. Representative trajectories in Age Residual between multiple tests are shown for 100 randomly selected participants. An improvement (younger) is colored in blue and a worsening in red (older).
**Figure S23:** Average number of tests and time between tests by supplement brand. This figure shows that the number of tests taken per participant is similar by supplement brand (A) and that the time between tests is also similar between PDL Health and TruMe (B). Please note that all participants in the PDL Health group in this analysis are on a dAKG subscription.
**Table S1:** Supplements, their synonyms, abbreviations and roots.
**Table S2:** Construction of health score.
**Table S3:** Linear model of health‐related behaviors. Multiple *R* ^2^: 0.02819, Adjusted *R* ^2^: 0.01757, *F*‐statistic: 2.654 on 31 and 2836 DF, *p*‐value: 0.000002024.
**Table S4:** Medication classes included in this manuscript. Please note that some of these classes were rarely used in our cohort and were thus dropped from the final analysis.
**Table S5:** Commonly consumed supplements. The table shows the number (count) and the fraction (frac) of participants that consume a certain supplement or supplement class. Only data for supplements consumed by more than 30 participants shown.
**Table S6:** Baseline characteristics of supplement users and non‐users.
**Table S7:** Baseline characteristics of dAKG users and non‐users.
**Table S8:** Linear model for dAKG effects. Multiple *R* ^2^: 0.0194, Adjusted *R* ^2^: 0.0103, *F*‐statistic: 2.12 on 23 and 2462 DF, *p*‐value: 0.00144. We quantified potential outliers using Cook's distance and leverage values from the fitted model, and defined potentially influential observations as those with Cook's distance greater than 4/n or leverage greater than twice the mean leverage. Observations meeting either of these criteria were flagged as influential for downstream analyses. A total of 355 outlier rows were dropped to improve kurtosis and skewness of the data. The model with and without outliers produced similar results.
**Table S9:** Mixed linear model for dAKG effects. Formula: marker ~ timepoint * (curcumin + Q10 + calcium + joint + dAKG_subscription + dAKG + baseline_bioage) + age_c + sex + smoking + general_health_status + date_diff_val + (1 | participant_id).

## Data Availability

Researchers who are interested in exploring this dataset can reach out to the principal investigator.
