# Supplementary material for: Supplements and Drugs Are Associated With Biological Age in a Cohort of Exceptionally Healthy Individuals
Source: Aging Cell. 2026 May 21;25(6):e70517. doi: 10.1111/acel.70517 (PMC13239866; doi:10.1111/acel.70517)
Supplement: Supplementary file 1 — Figure S1: Subject recruitment flowchart. Most participants bought a TruMe epigenetic test online. Some participants recruited through PDL Health received a gift voucher for a free test. Please note that not all dAKG supplement users were recruited through PDL Health. Conversely, not all who were recruited via PDL Health necessarily took or declared taking dAKG supplement at the time of the survey. Figure S2: Comparing methods for data cleaning. We show here that two methods of cleaning and counting supplements produce very similar total counts for supplements. In method 1, common typographic mistakes are corrected by hand while in method 2, all mistakes are automatically corrected using ChatGPT o3. Figure S3: Percentage of “rare” and “common” supplements taken by participants. Rare supplements here are defined as all supplements that are not exclusively containing vitamins, minerals, omega‐3 fatty acids and are not joint support supplements. These supplements have been rarely studied in prior studies. Figure S4: Age Residual vs chronological age. The mean Age Residual is stable across different age strata. Figure S5: Age Residual by recruiter (supplement brand or clinic). The Age Residual for participants recruited via different companies, longevity clinics and supplement brands varies moderately and non‐significantly. Showing brands with more than 40 data points. N = 3641. Figure S6: Age Residual by ethnicity. The Age Residual for participants across different ethnicities is comparable. Figure S7: Age Residual by country. We observe significant differences in Age Residual across participants from different countries in our cohort. Figure S8: Traditional risk factors are associated with biological age. (A) Smoking is associated with significantly higher Age Residual. (B) Restful sleep is associated with non‐significantly lower Age Residual. (C) Better health status is associated with lower Age Residual. (D) Comparison of participants in the lowest and highest decil [file ACEL-25-e70517-s001.docx]

**
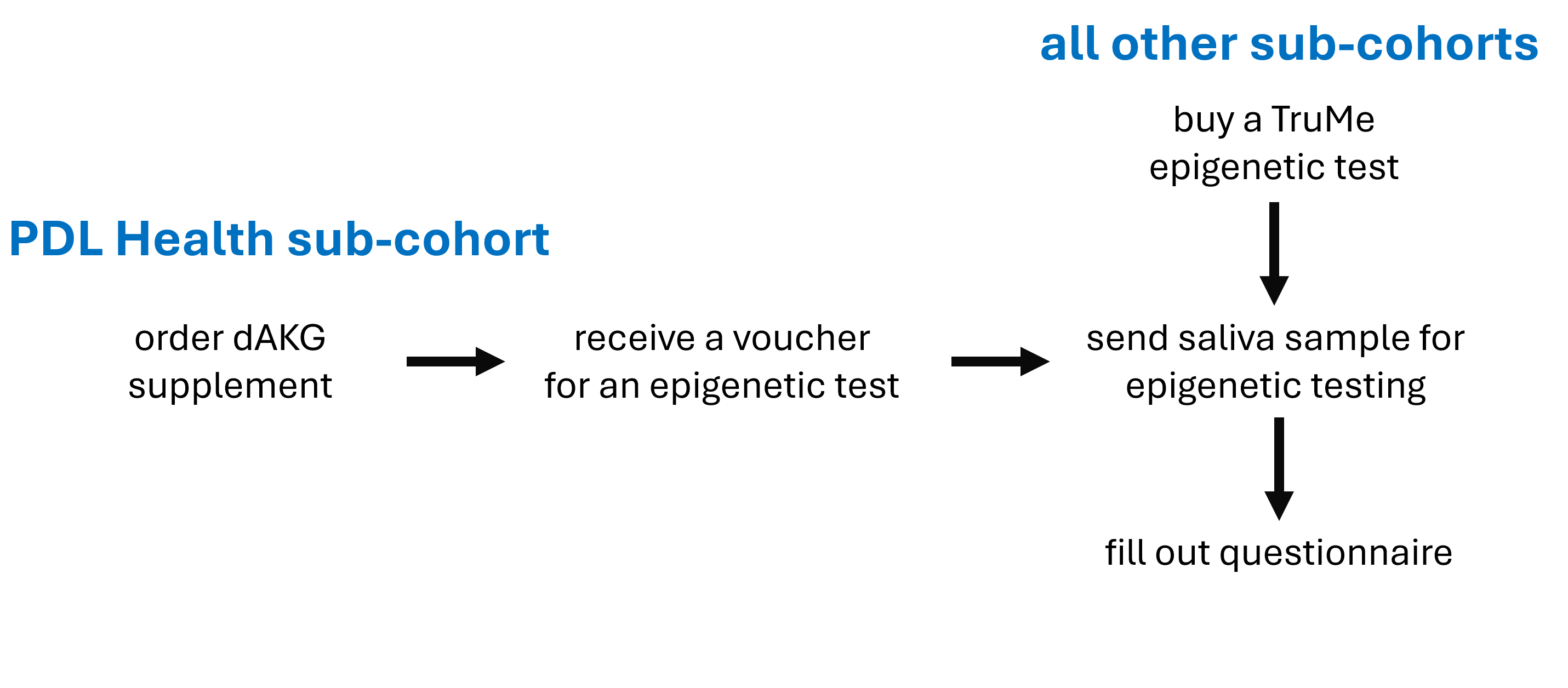
 Figure S1. Subject recruitment flowchart**Most participants bought a TruMe epigenetic test online. Some participants recruited through PDL Health received a gift voucher for a free test. Please note that not all dAKG supplement users were recruited through PDL Health. Conversely, not all who were recruited via PDL Health necessarily took or declared taking dAKG supplement at the time of the survey.


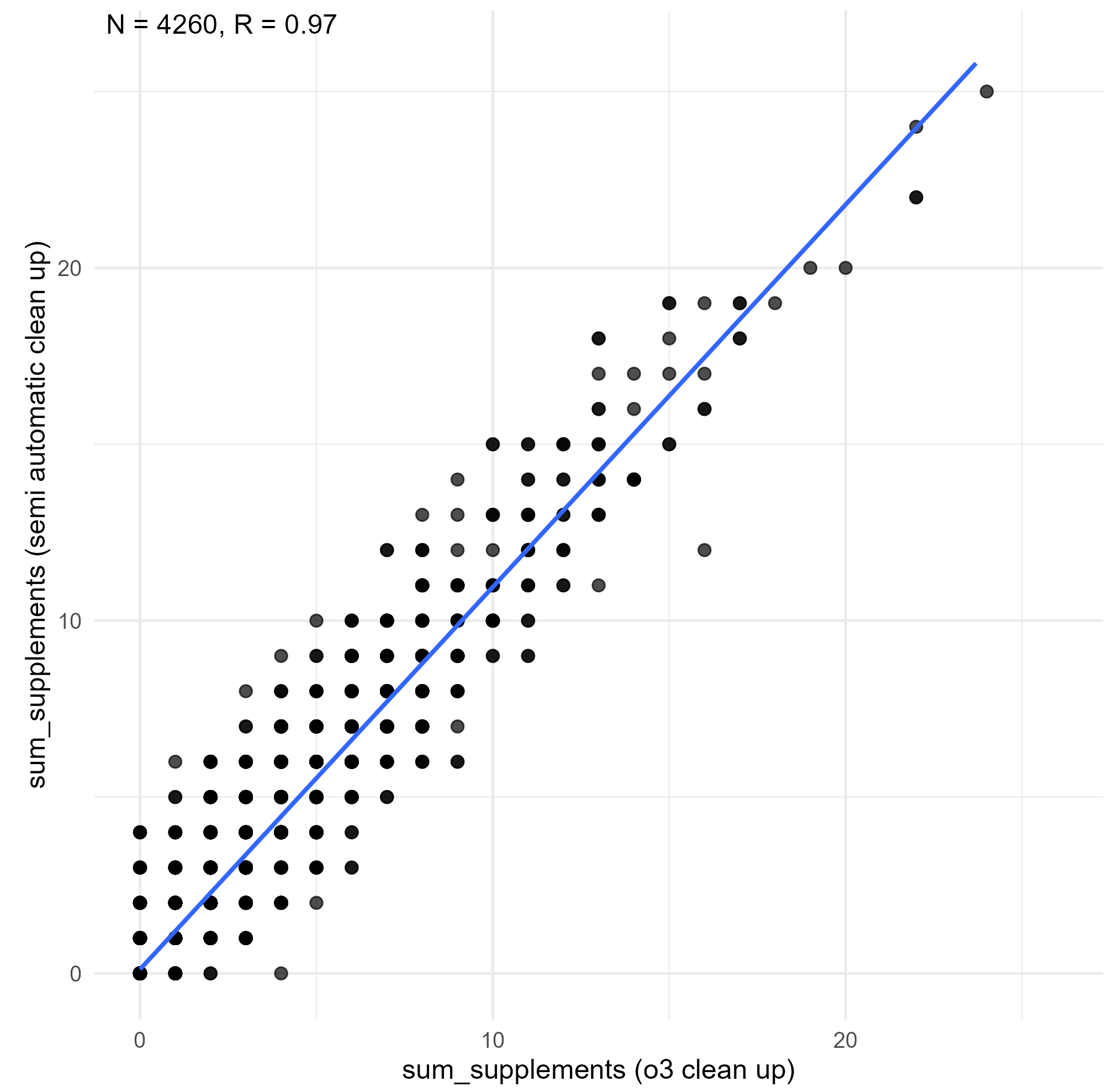
 **Figure S2. Comparing methods for data cleaning**We show here that two methods of cleaning and counting supplements produce very similar total counts for supplements. In method 1, common typographic mistakes are corrected by hand while in method 2, all mistakes are automatically corrected using ChatGPT o3.


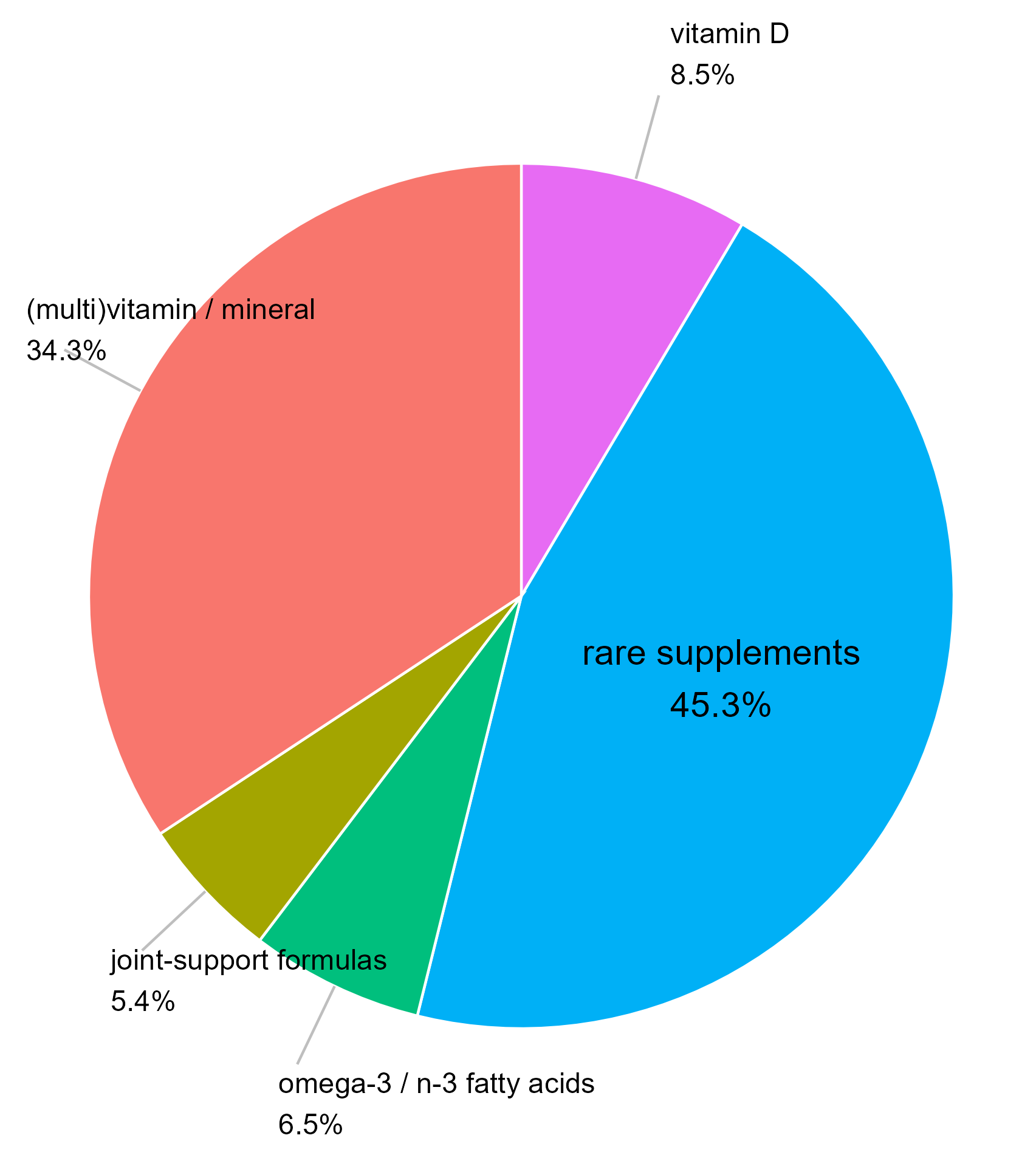

**Figure S3. Percentage of “rare” and “common” supplements taken by participants**Rare supplements here are defined as all supplements that are not exclusively containing vitamins, minerals, omega-3 fatty acids and are not joint support supplements. These supplements have been rarely studied in prior studies.


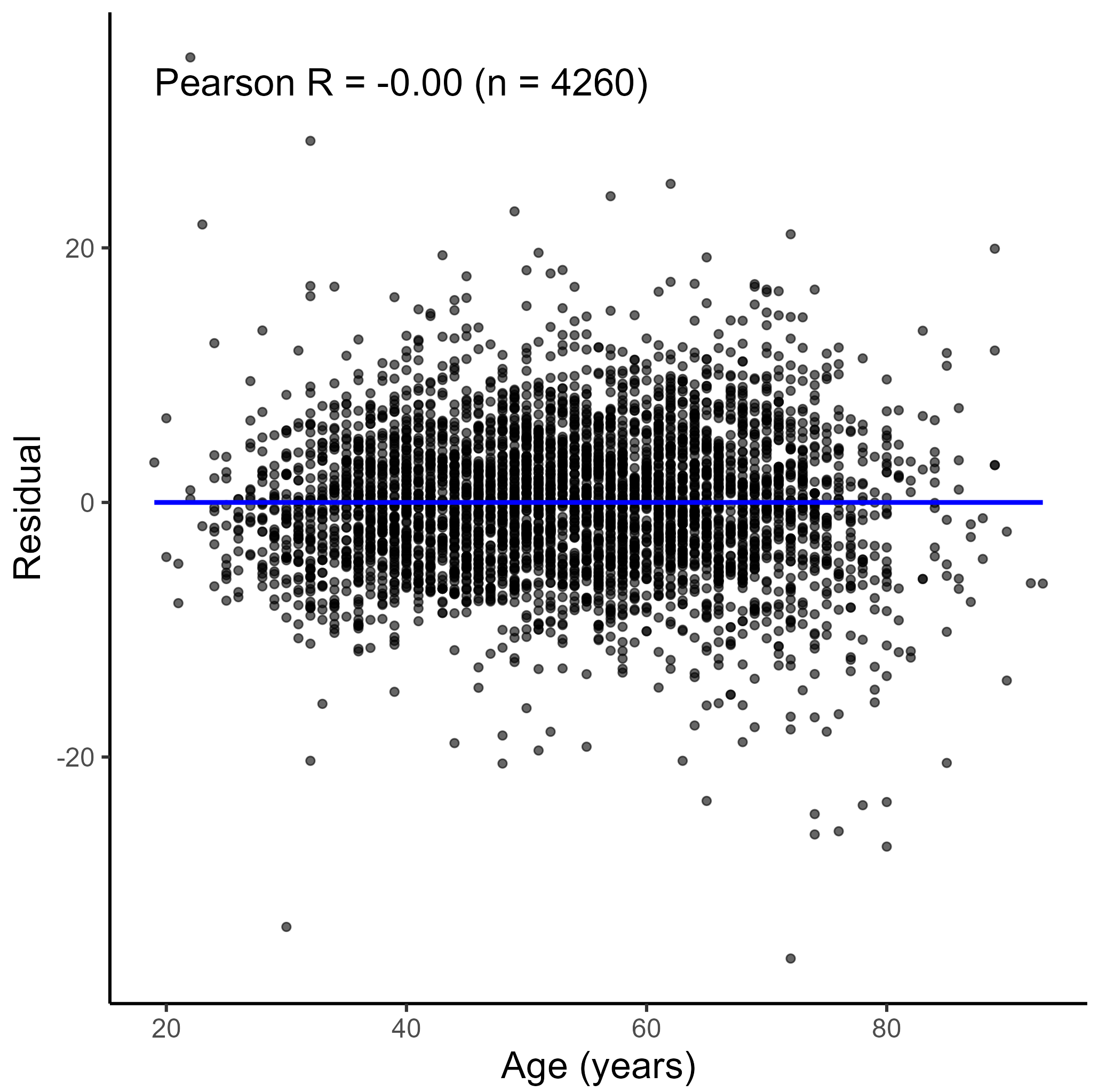
 **Figure S4. Age Residual vs chronological age**The mean Age Residual is stable across different age strata.


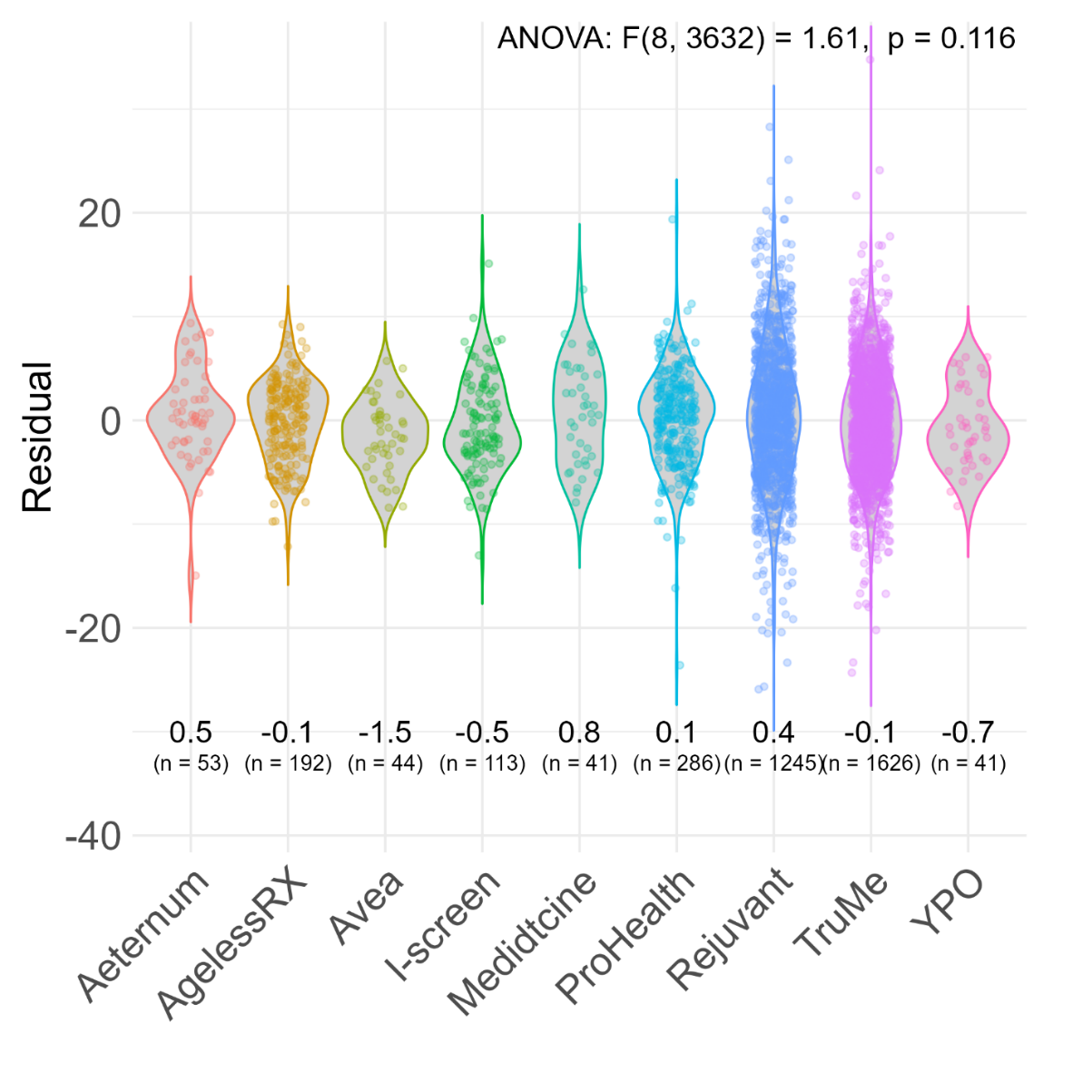


**Figure S5. Age Residual by recruiter (supplement brand or clinic)**The Age Residual for participants recruited via different companies, longevity clinics and supplement brands varies moderately and non-significantly. Showing brands with more than 40 data points. N=3641.


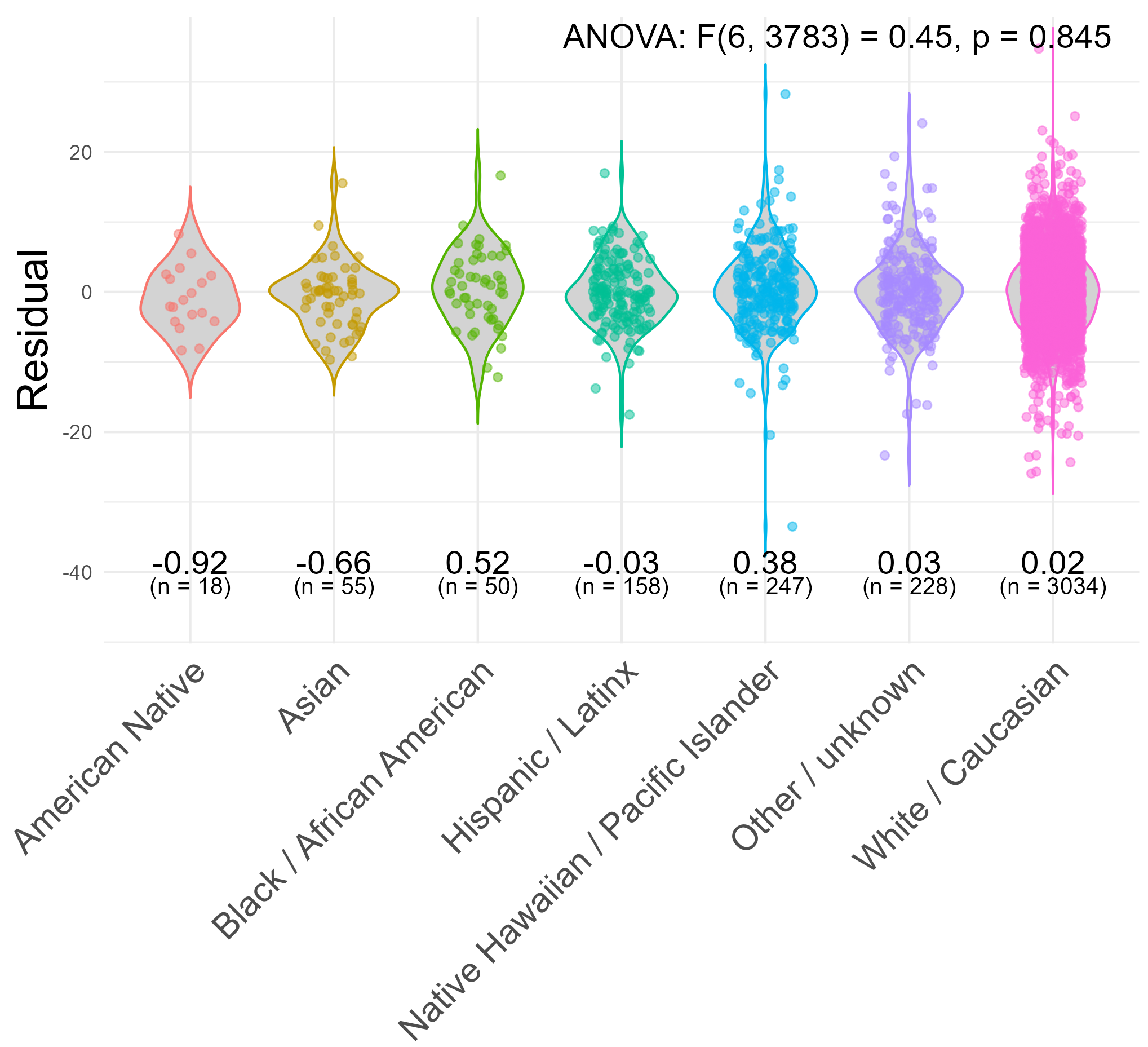

**Figure S6. Age Residual by ethnicity**
The Age Residual for participants across different ethnicities is comparable.


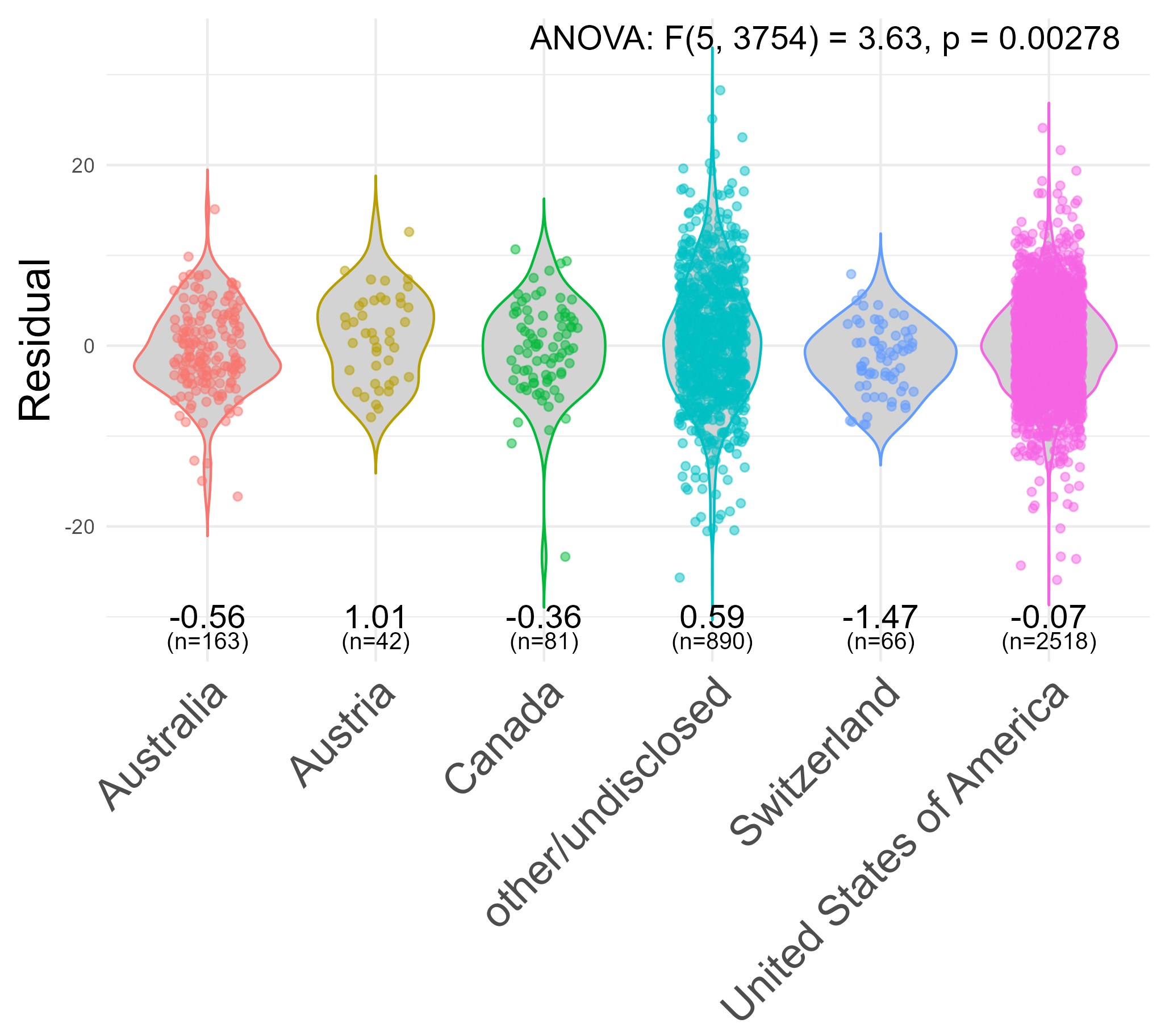


**Figure S7. Age Residual by country**We observe significant differences in Age Residual across participants from different countries in our cohort.


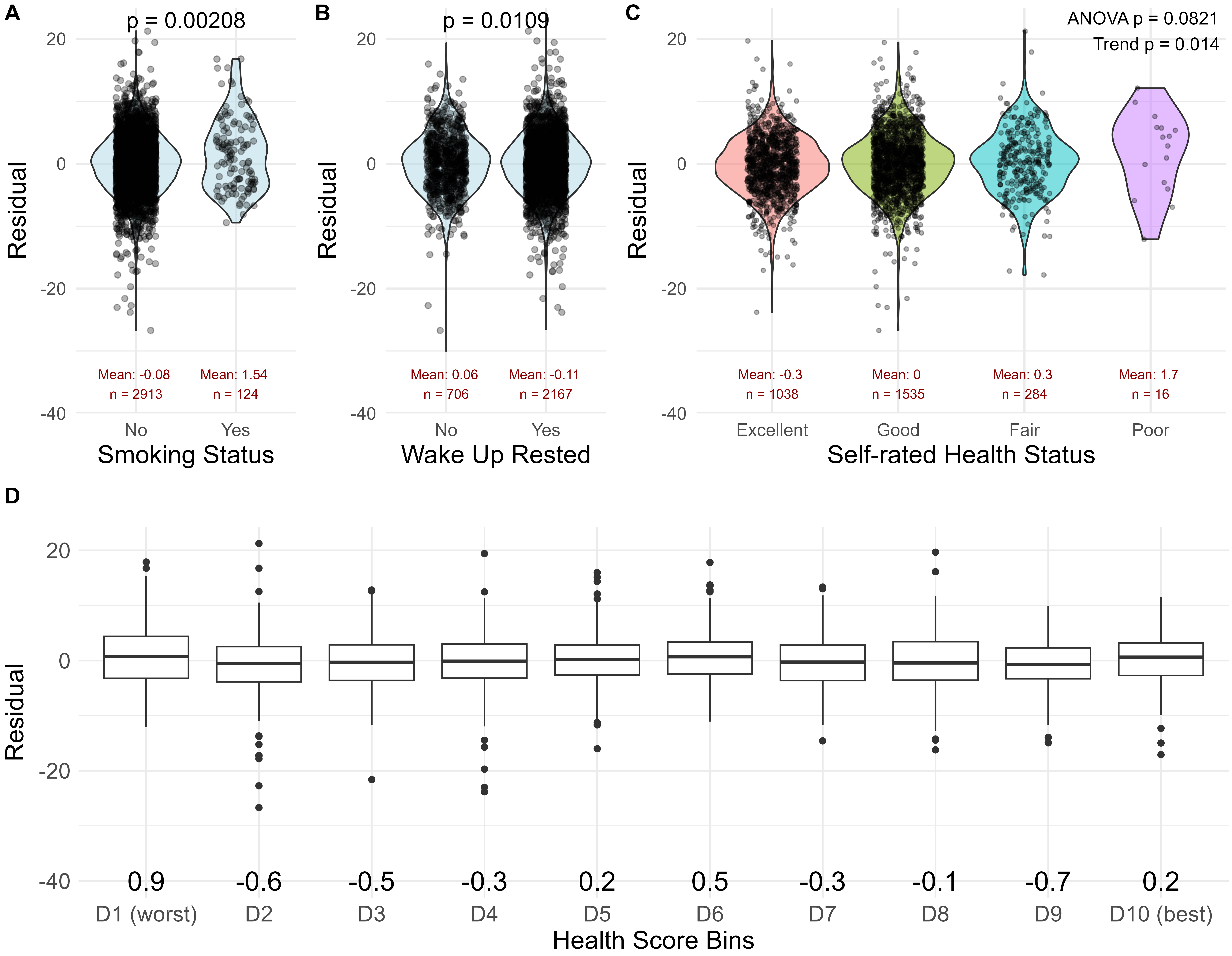
 **Figure S8. Traditional risk factors are associated with biological age**
(A) Smoking is associated with significantly higher Age Residual.
(B) Restful sleep is associated with non-significantly lower Age Residual.
(C) Better health status is associated with lower Age Residual.
(D) Comparison of participants in the lowest and highest decile of our health score that includes general health status, general mental health status, rested on waking, weekly exercise frequency, smoking status, supplement use, drug use, alcohol intake and perceived stress level.


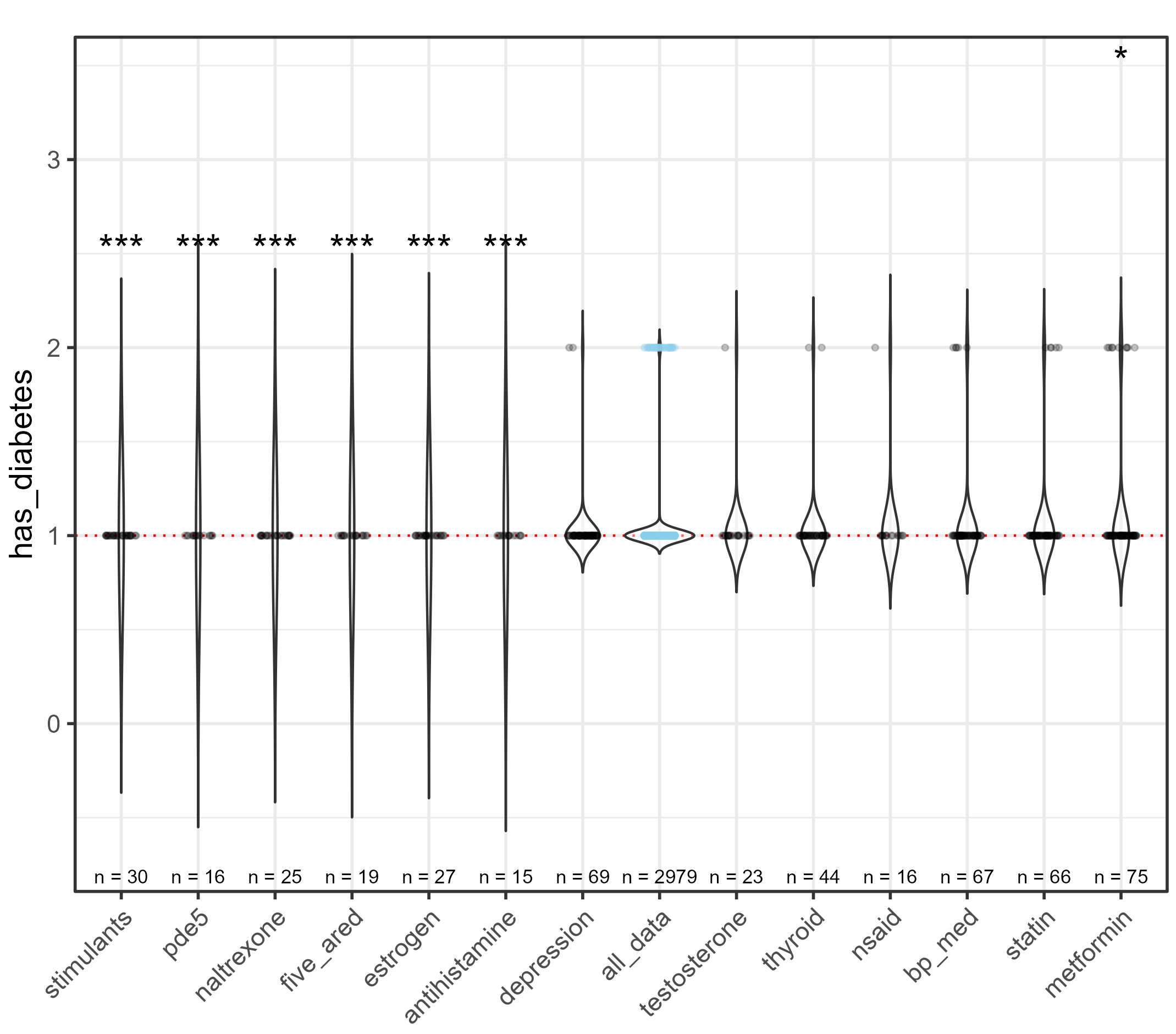
 **Figure S9. Different medications are associated with diabetes prevalence**Here we show the prevalence of diabetes (“has diabetes” coded as 2) across different medication classes. Corrected for multiple testing using Benjamini–Hochberg. N≥15 per group. stimulants = central nervous system stimulants, nsaid = non-steroidal anti-inflammatory drugs, ppi_antacid = proton-pump inhibitors / antacids, naltrexone = opioid receptor antagonists, benzo = benzodiazepines, bp_med = antihypertensive agents, glp1 = glucagon-like peptide-1 receptor agonists, depression = antidepressants, antithrombosis = antithrombotic agents, five_ared = 5α-reductase inhibitors, pde5 = phosphodiesterase-5 inhibitors, rapa = rapamycin, thyroid = thyroid hormone replacement therapy.


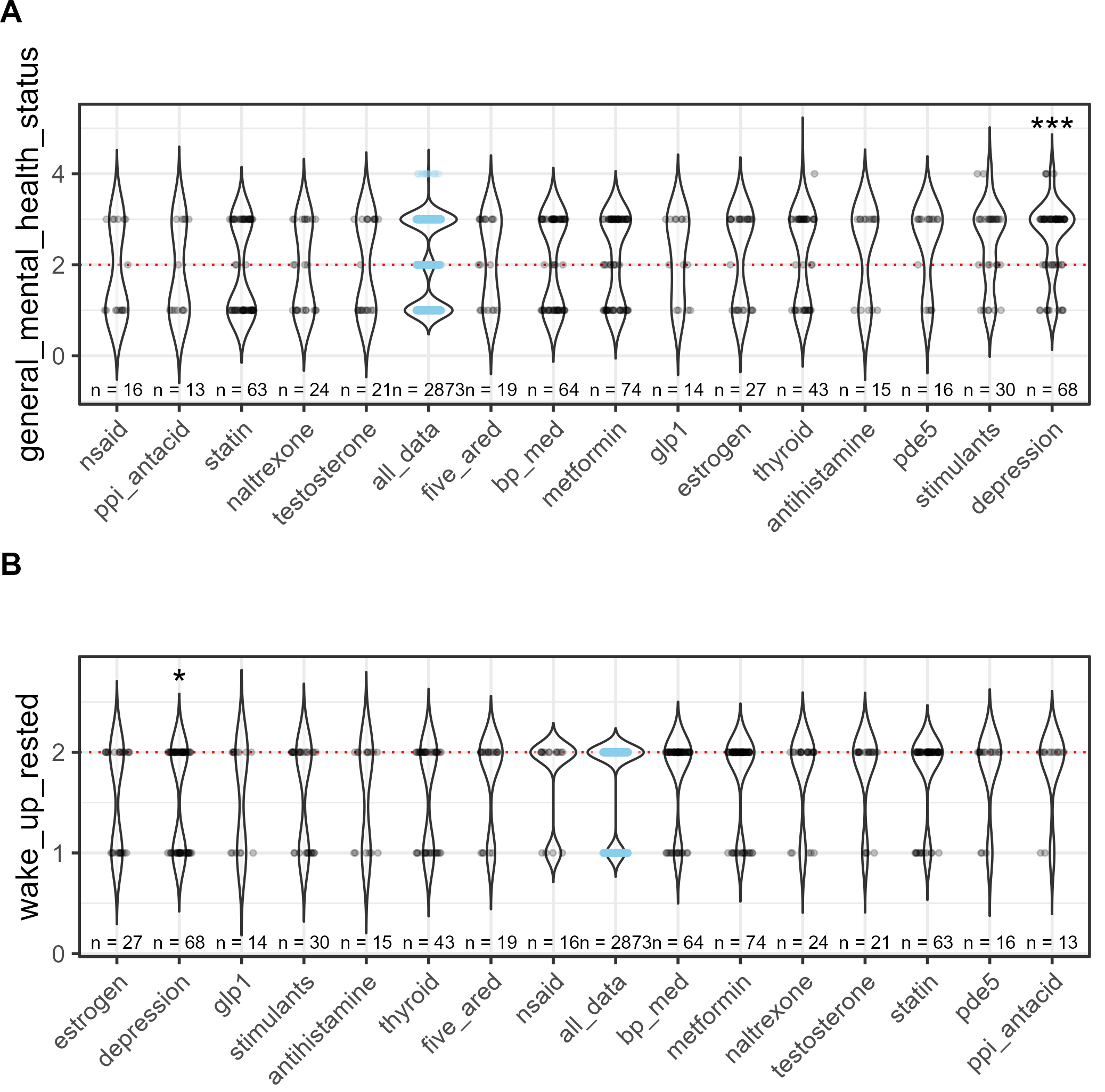

**Figure S10. Antidepressants use is associated with mental health and sleep quality**(A) Antidepressant use is associated with significantly worse mental health scores. A higher score means worse mental health.
(B) Antidepressant use is associated with significantly less restful sleep. 1=waking up tired, 2=waking up rested.
Data was corrected for multiple testing using Benjamini–Hochberg. N≥15 per group. stimulants = central nervous system stimulants, nsaid = non-steroidal anti-inflammatory drugs, ppi_antacid = proton-pump inhibitors / antacids, naltrexone = opioid receptor antagonists, benzo = benzodiazepines, bp_med = antihypertensive agents, glp1 = glucagon-like peptide-1 receptor agonists, depression = antidepressants, antithrombosis = antithrombotic agents, five_ared = 5α-reductase inhibitors, pde5 = phosphodiesterase-5 inhibitors, rapa = rapamycin, thyroid = thyroid hormone replacement therapy.


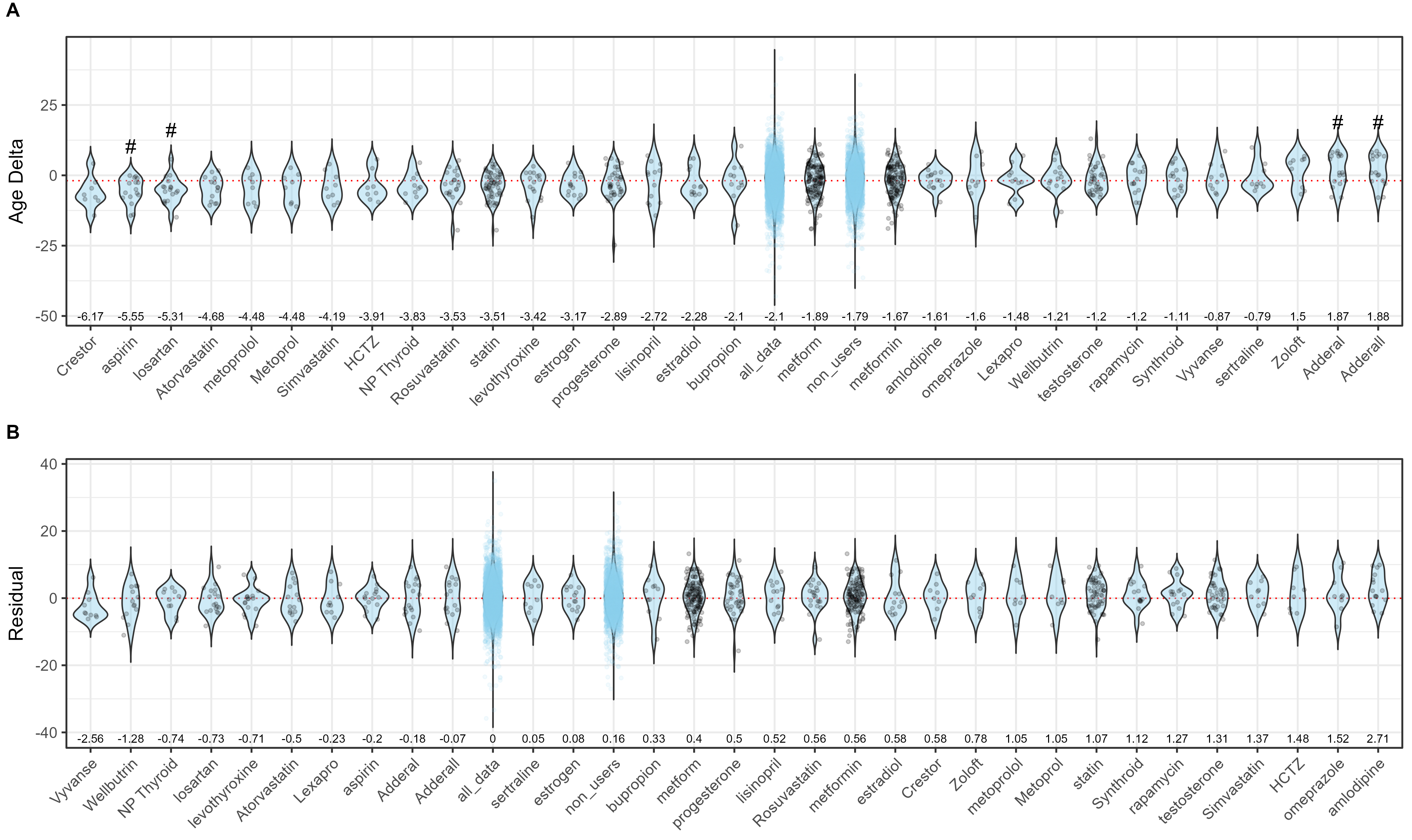
**Figure S11. Association between individual medications and measures of biological age**The impact of different drugs on Age Delta is shown in (A) and the impact of different drugs on Age Residual in (B). The list of drugs includes commonly used drug names and abbreviations.
The median of the control group (all_data) is indicated with a dashed red line. Control group and drug non-users (non_users) are plotted for comparison in light blue.
* and # indicate groups with a p<0.05 or p<0.10, respectively, after Benjamini–Hochberg correction when compared with all participants. Mean biological age shown on top of the x axis for each group.


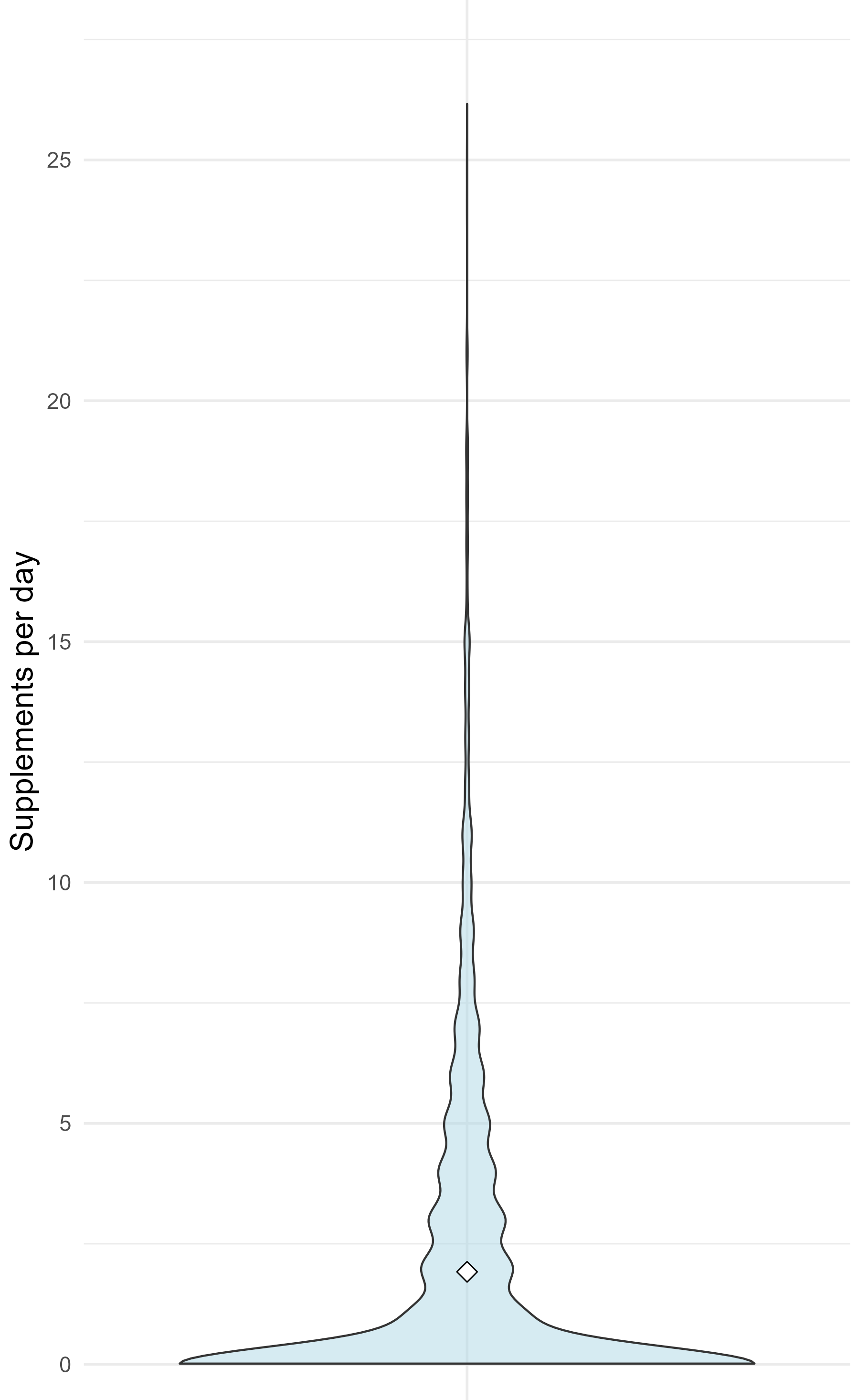

**Figure S12. Distribution of supplement consumption in our cohort**Most participants consume between zero and three supplements with a long tail of high consumers.


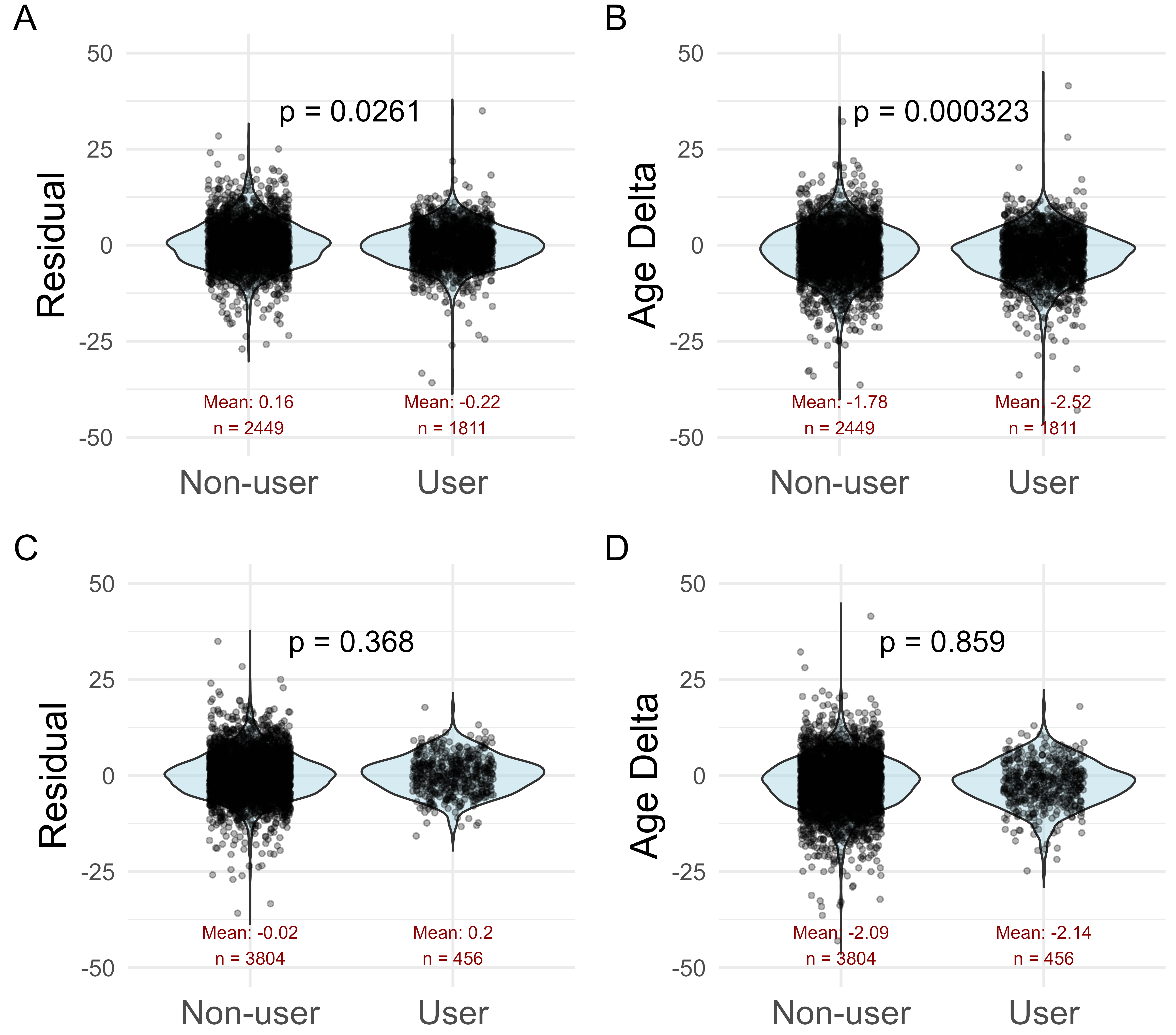


**Figure S13. Impact of supplements and medications on biological age**
Supplement users have significantly lower Age Residual (A) and Age Delta (B). In contrast, those who list using medications on the survey do not show reduced biological age measures (A, B). N=3790.


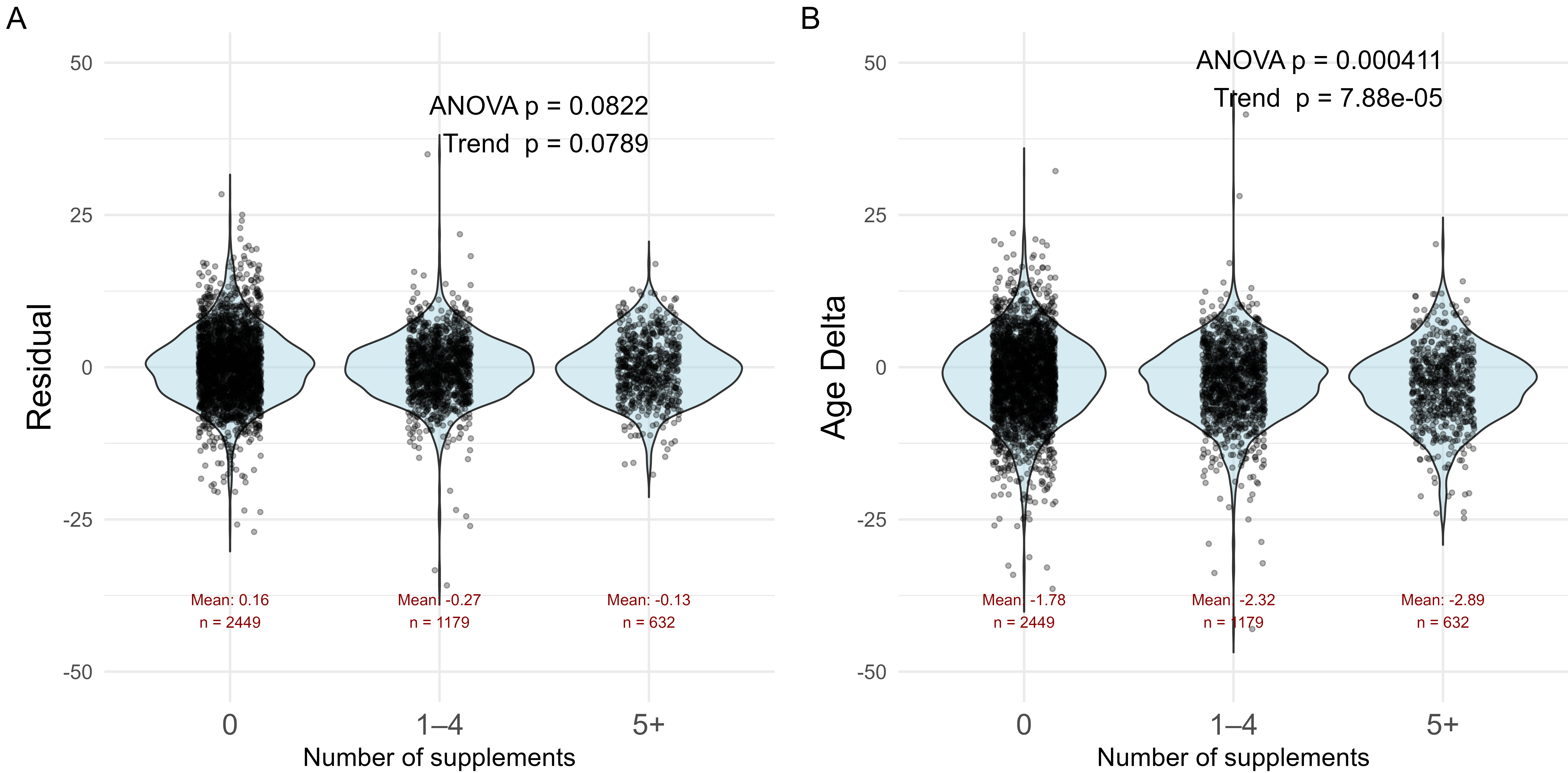
 **Figure S14. Heavy supplement use is not associated with adverse effects on biological age**Heavy supplement users (5+ different supplements taken) have similar Age Residual (A) but lower Age Delta (B) compared with non-users and moderate users.


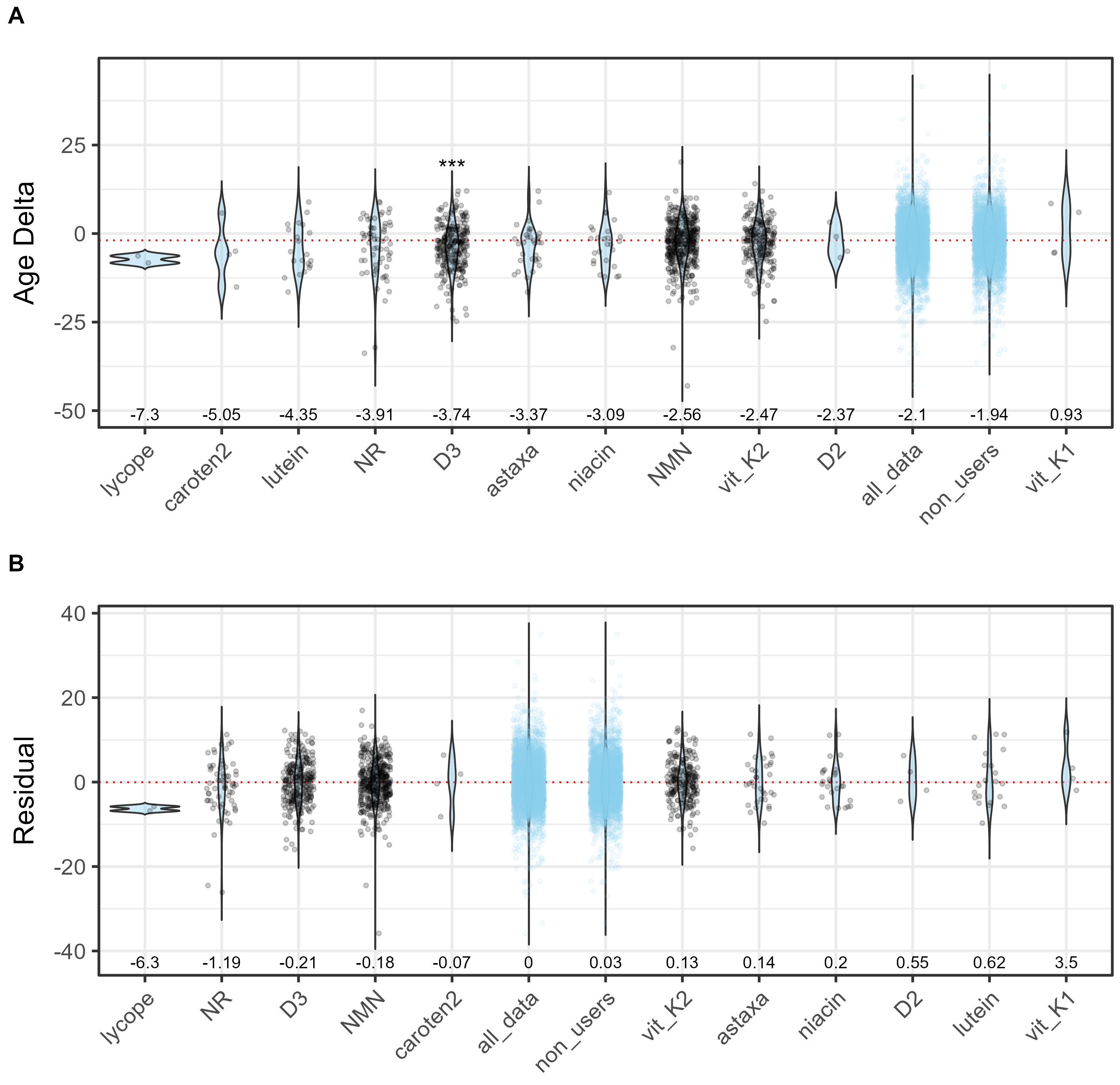
 **Figure S15. Impact of supplements on biological age**
Certain supplements are associated with lower biological age. The median of the control group (all_data) is indicated with a dashed red line. Control group and all supplement non-users (non_users) are plotted for comparison in light blue. *,**, *** indicates groups with a p<0.05, p<0.01 or p<0.001 after Benjamini–Hochberg correction when compared with all participants. Mean biological age shown on top of the y axis for each group (lower Age Delta or Residual means younger than expected). lycope = lycopene; NR = nicotinamide riboside; D3 = vitamin D3 (cholecalciferol); NMN = nicotinamide mononucleotide; caroten2 = β-carotene; vit_k2 = vitamin K2 (menaquinone); astaxa = astaxanthin; niacin = niacin (vitamin B3); D2 = vitamin D2 (ergocalciferol); lutein = lutein; vit_k1 = vitamin K1 (phylloquinone).


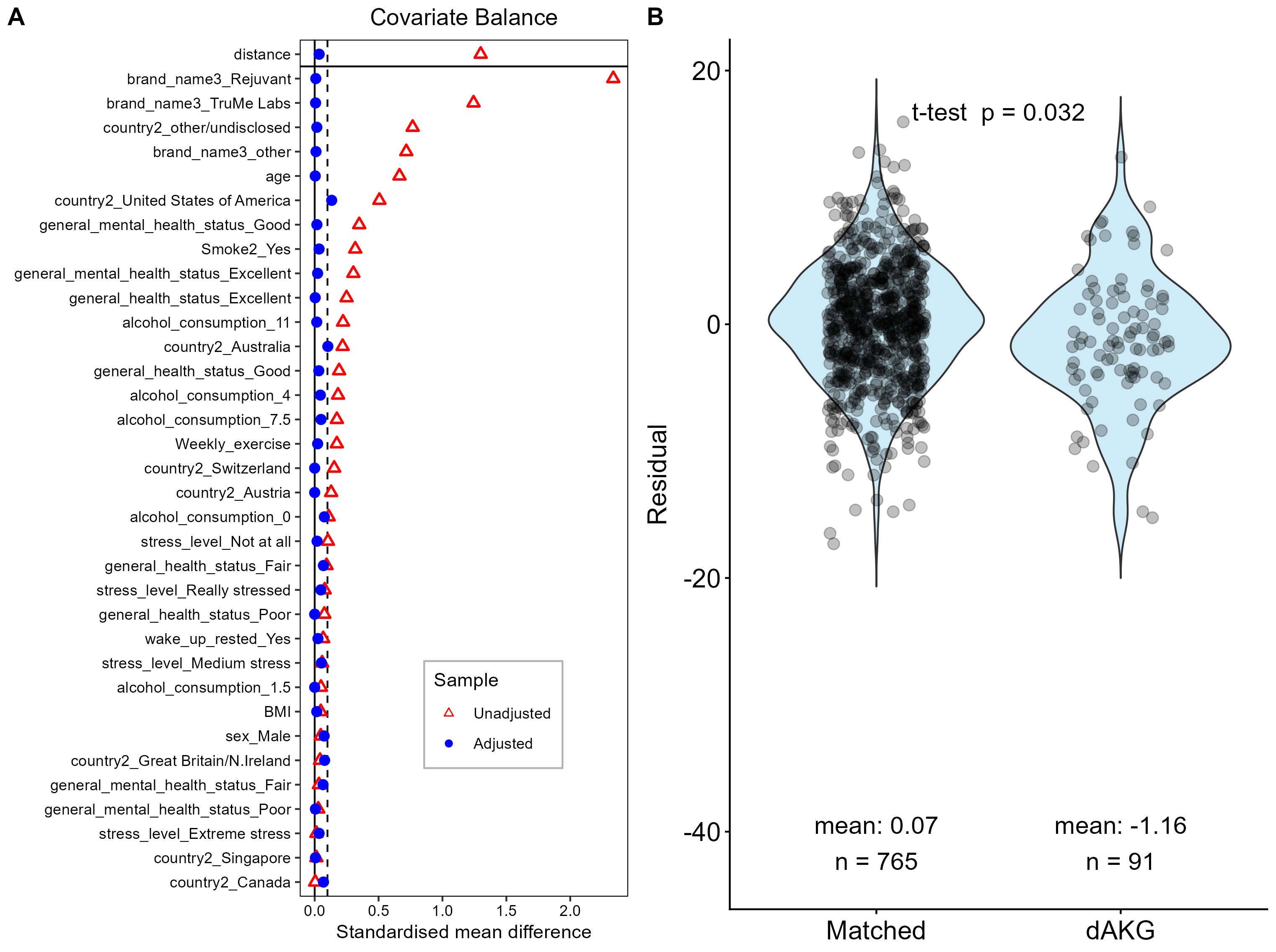


**Figure S16. dAKG users show a lower biological age compared to non-users**Matching balances covariate distribution across groups (A). dAKG is associated with lower Age Residual even when compared to matched controls (B).


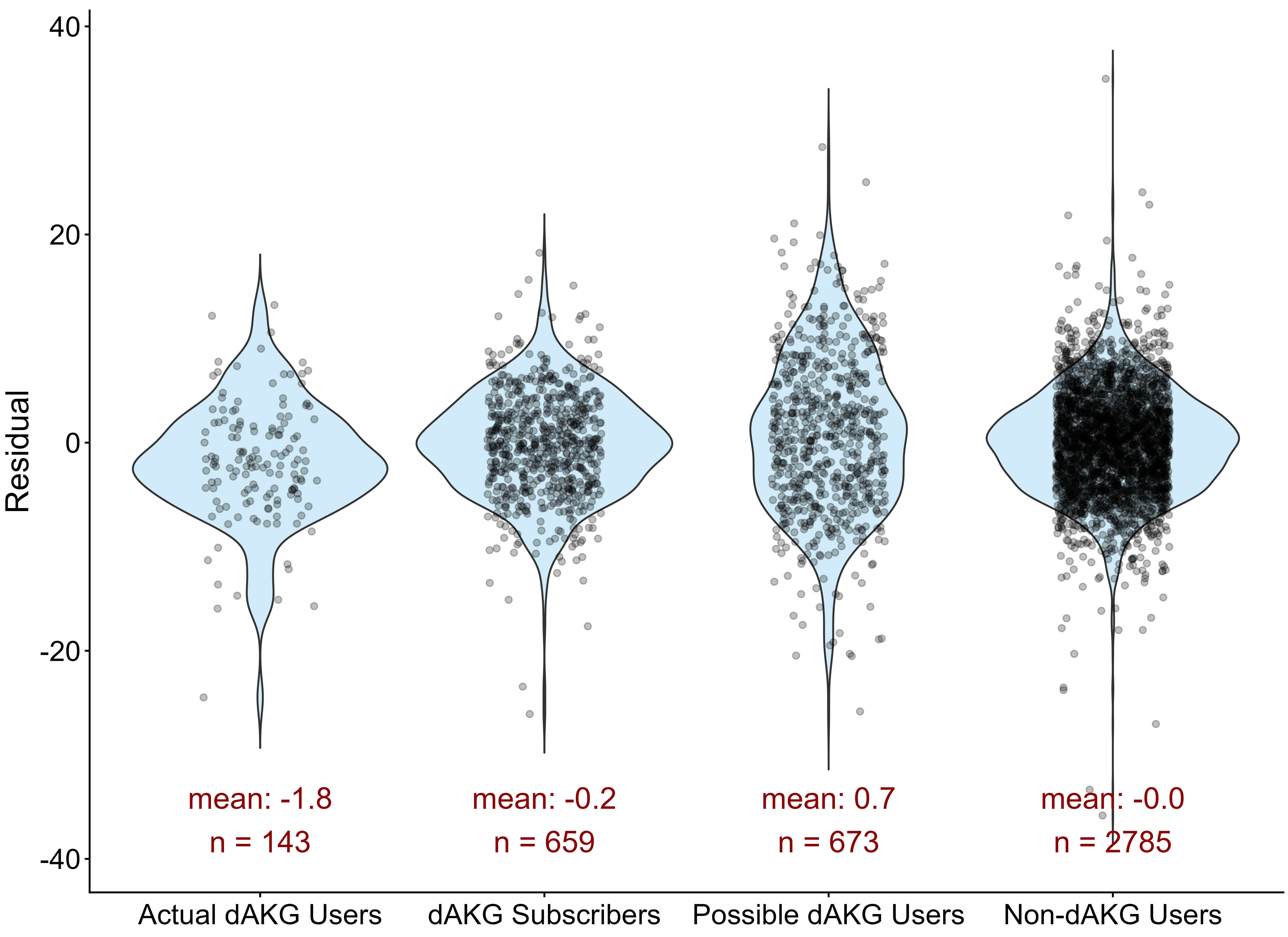


**Figure S17. dAKG subscribers show a similar biological age to non-users**Actual dAKG users show the lowest Age Residual while dAKG subscribers are closer to non-users. Actual dAKG users are those who mention dAKG in the supplement questionnaire, dAKG subscribers have a subscription plan, while possible dAKG users bought dAKG at one point in time. Non-dAKG users includes all participants that fit neither of those three categories.


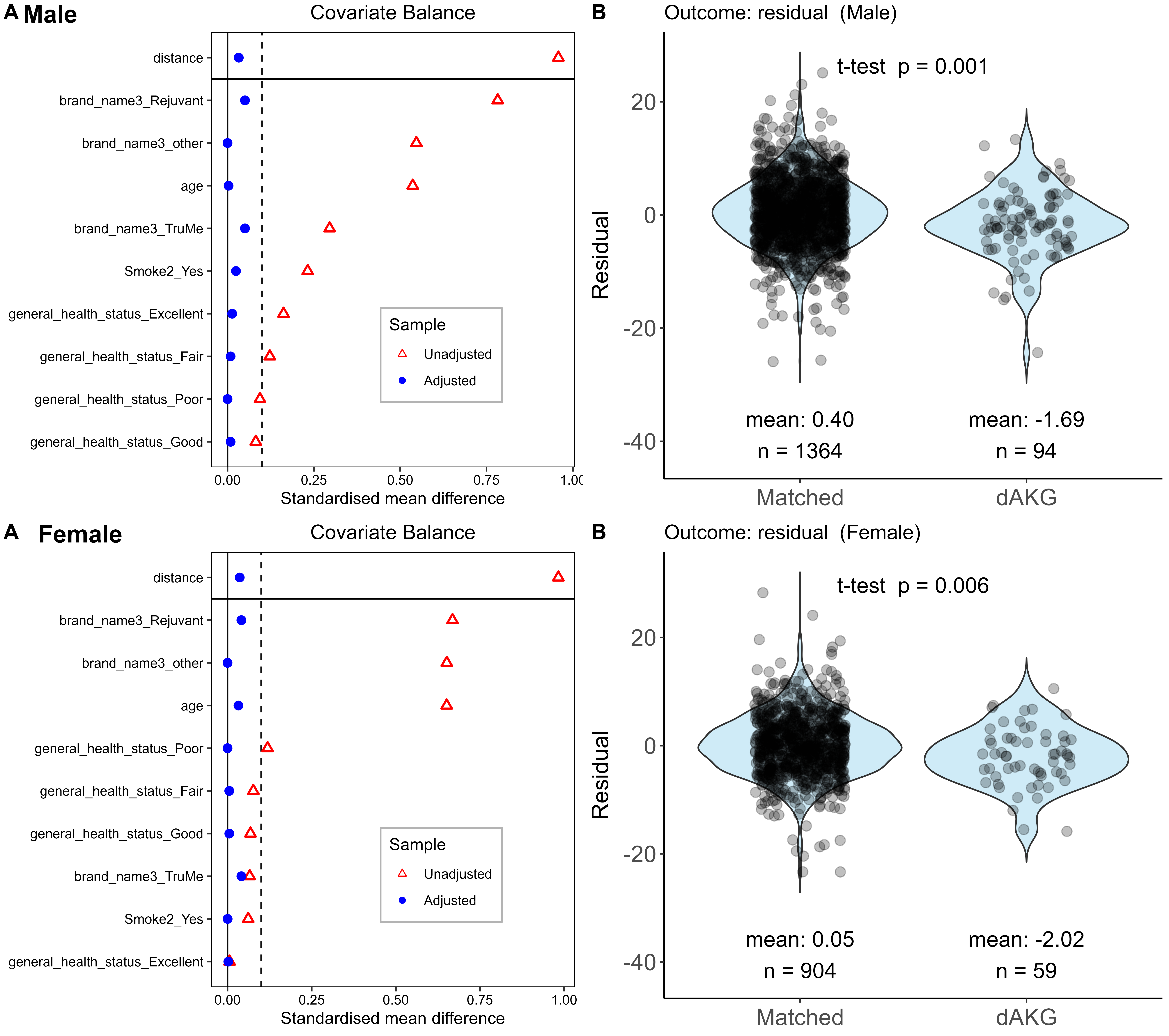
**Figure S18. dAKG effects on biological age in men and women**After adjusting for age, smoking, general health status and brand we find that dAKG is associated with significantly lower Age Residual in men (A) and women (B).


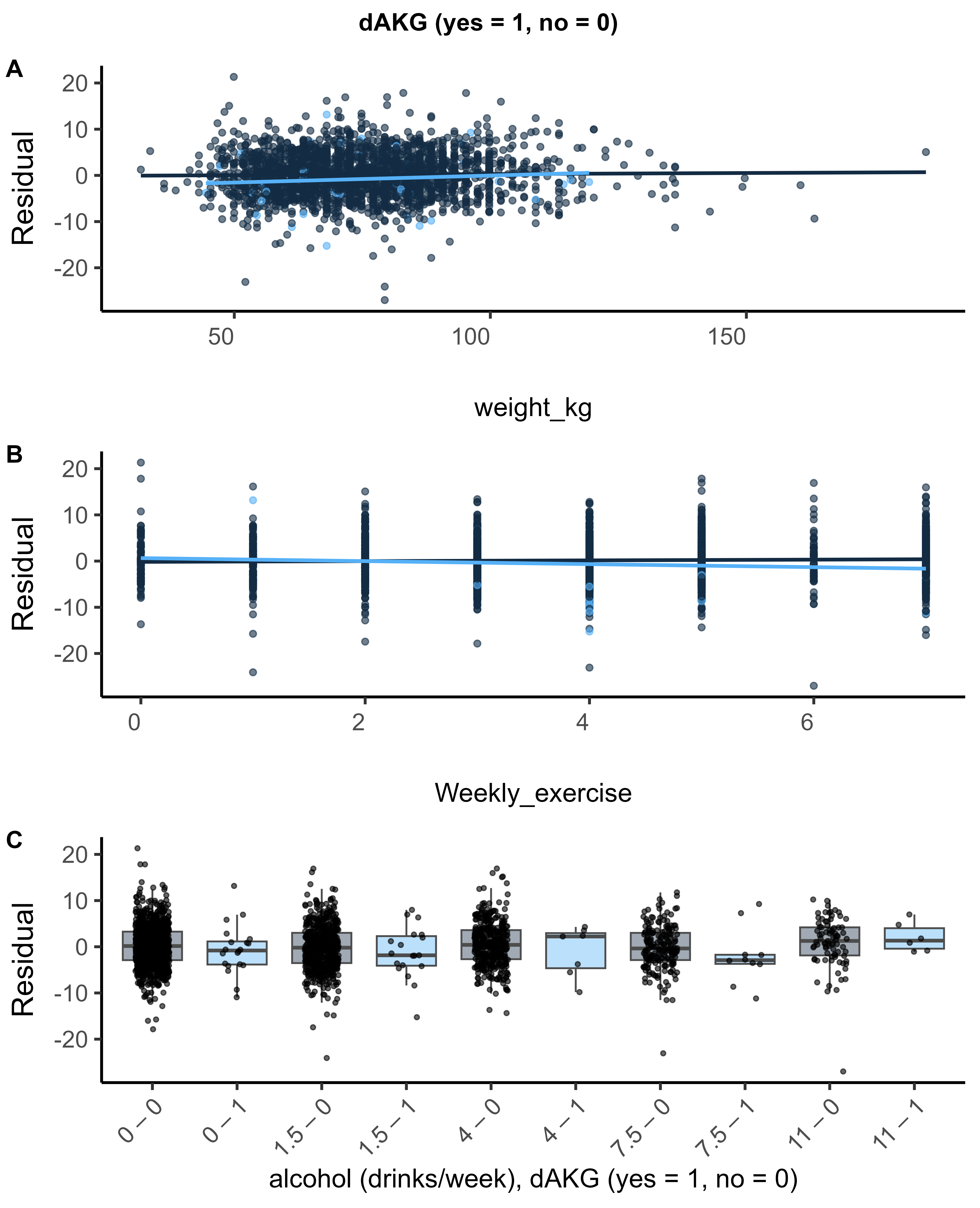
 **Figure S19. The effects of dAKG on biological age in different subgroups**dAKG use (in blue) is associated with decreased Age Residual regardless of weight (A), weekly exercise volume (B) and amount of alcohol consumed (C). Those who exercise regularly might benefit more from dAKG.


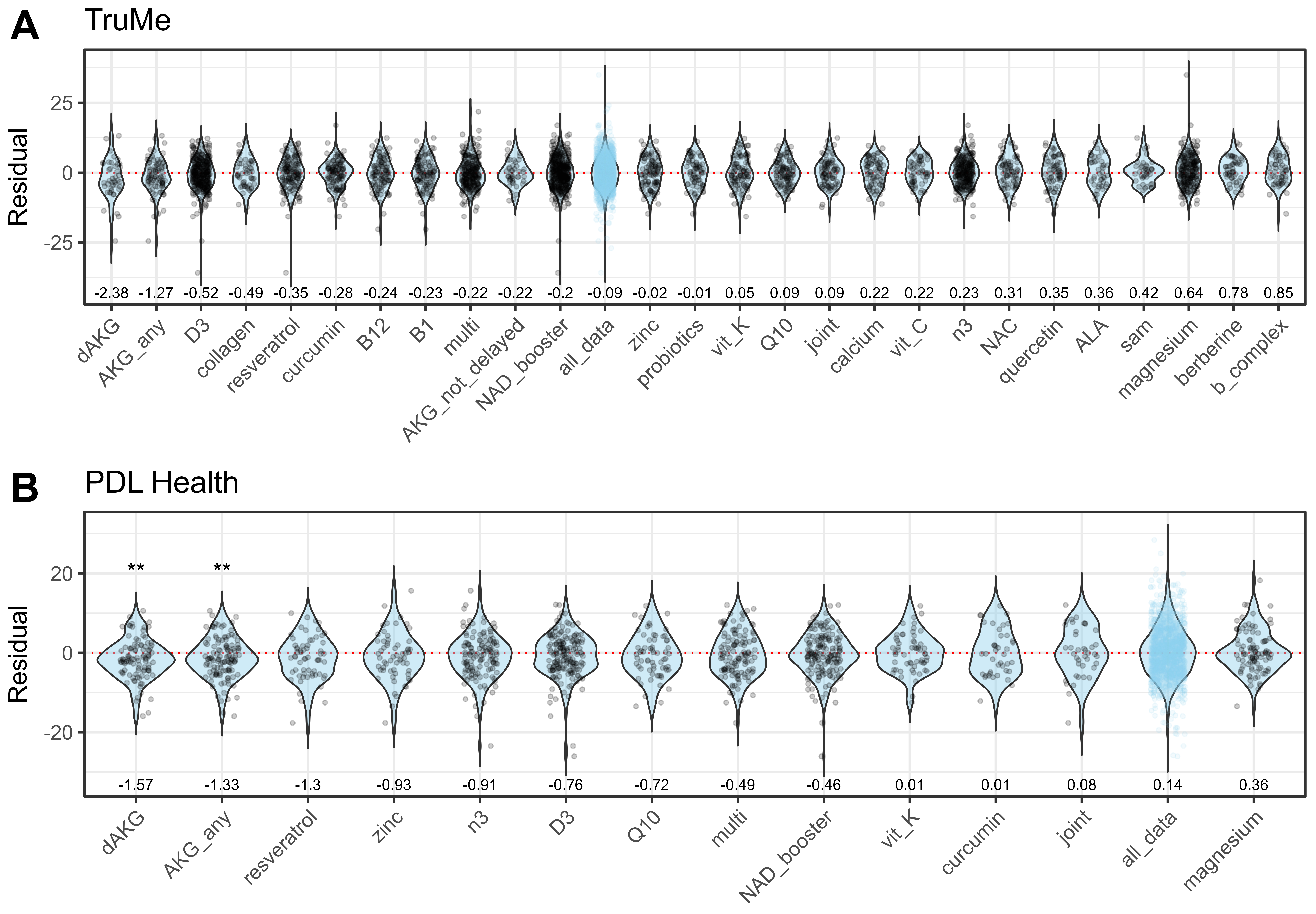


**Figure S20. dAKG is associated with lower biological age in two different sub-cohorts**Only supplements with more than 40 users are shown in the figure.
(A) dAKG supplement lowers Age Residual in the TruMe sub-cohort (p_unadj=0.036, p_adj=0.53).
(B) dAKG supplement significantly lowers Age Residual in the PDL Health sub-cohort (p_unadj=0.00072, p_adj=0.0096).


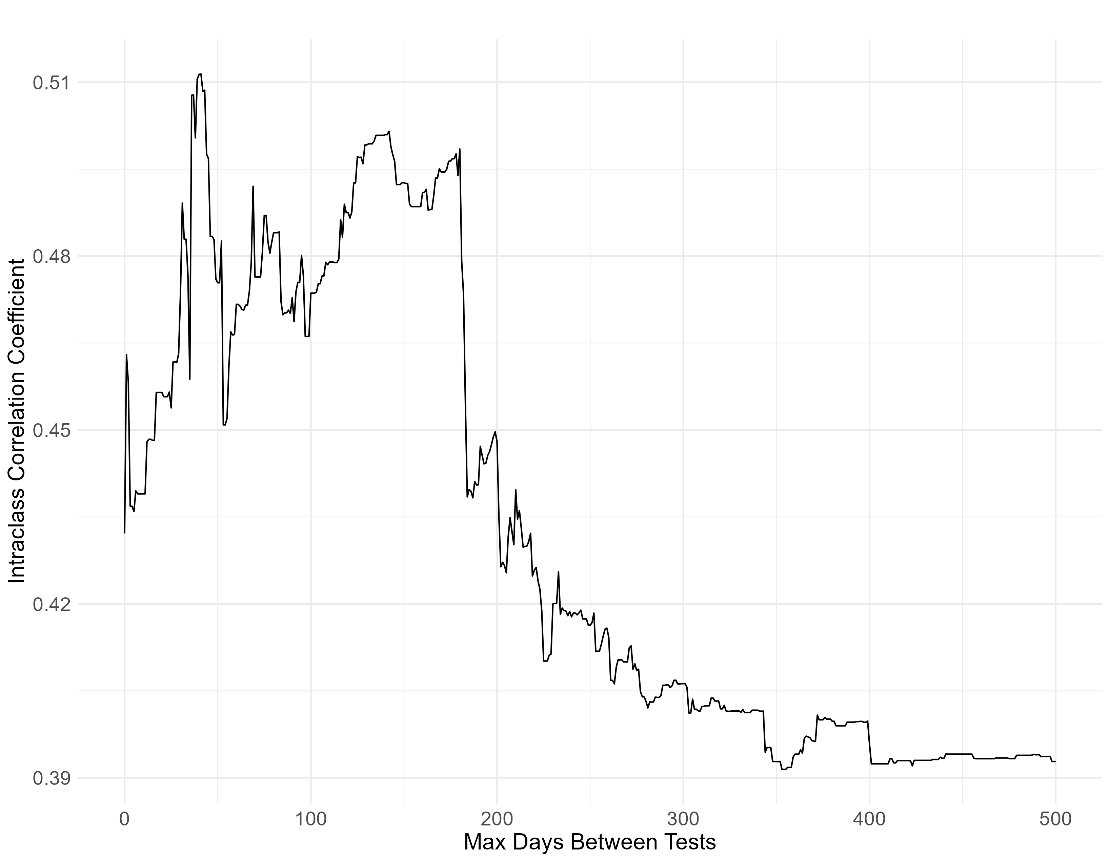
 **Figure S21. Test to test variability of the saliva epigenetic clock**
The intraclass correlation coefficient (ICC) is calculated against a running cut-off. We calculate the ICC for all participants that had two epigenetic tests less than X days apart (“Max Days Between Tests” shown on the X-axis). As expected, the higher the time elapsed between tests the lower the correlation between two given tests on average. Fluctuations, especially, at earlier time points may be due to low sample sizes.


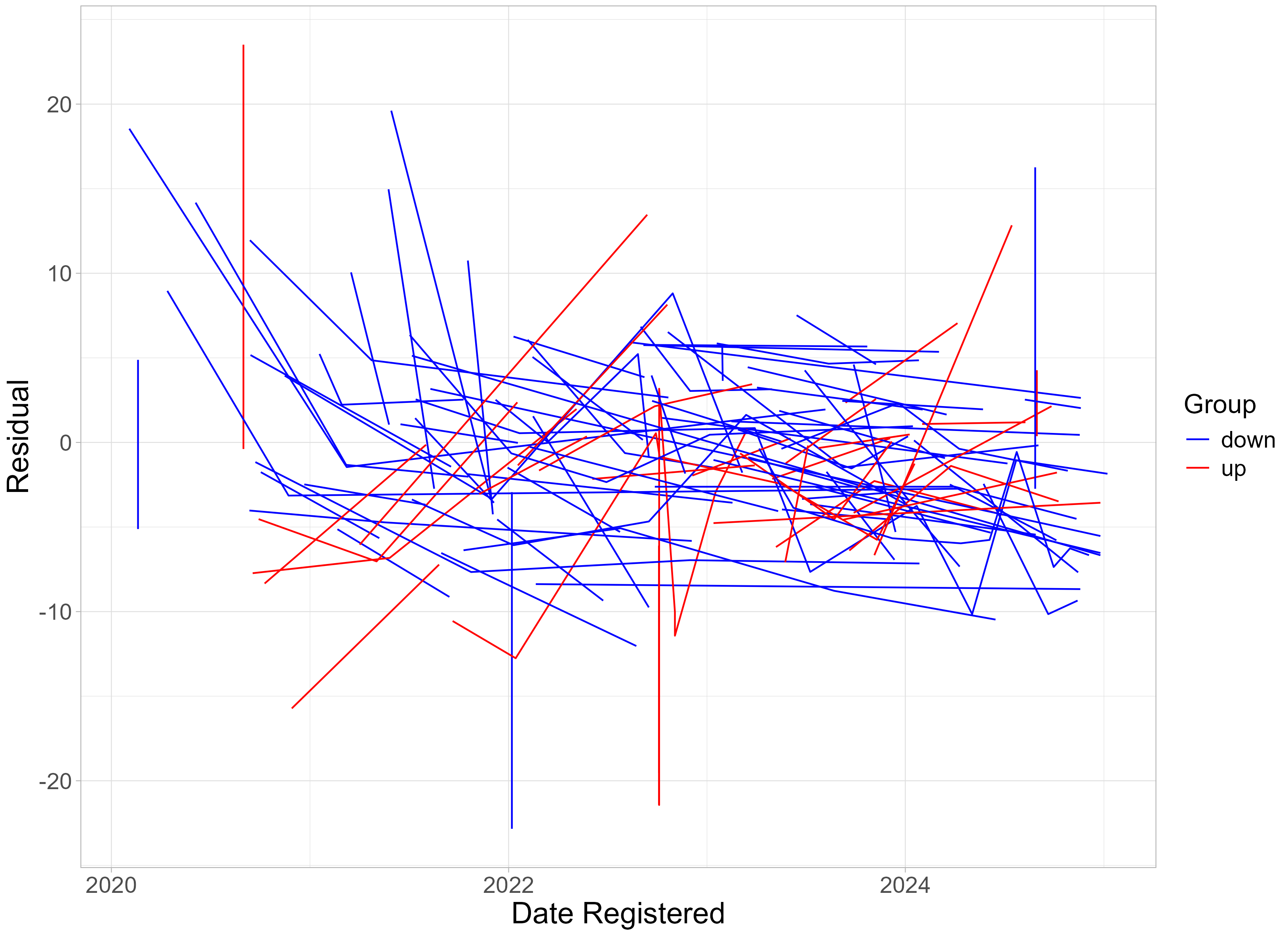


**Figure S22. Biological age trajectories for a random subset of participants**Representative trajectories in Age Residual between multiple tests are shown for 100 randomly selected participants. An improvement (younger) is colored in blue and a worsening in red (older).


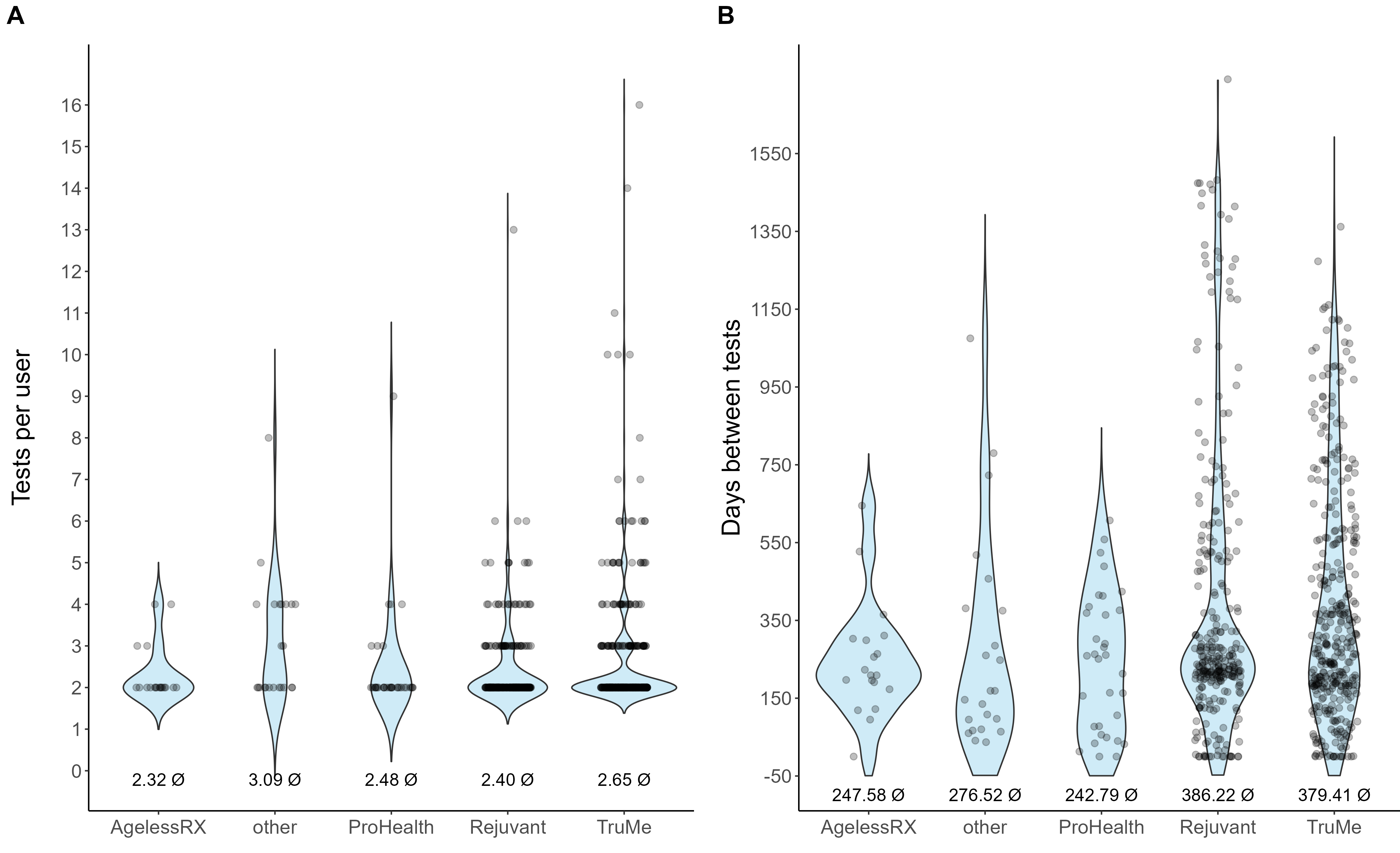

**Figure S23. Average number of tests and time between tests by supplement brand**
This figure shows that the number of tests taken per participant is similar by supplement brand (A) and that the time between tests is also similar between PDL Health and TruMe (B). Please note that all participants in the PDL health group in this analysis are on a dAKG subscription.

| ID | supplement | supplement_synonyms |
| --- | --- | --- |
| 1 | AKG_any | rejuvant, akg, ketoglutarate |
| 2 | AKG_not_delayed | akg, ketoglutarate |
| 3 | ALA | ala, lipoic acid |
| 4 | ALCAR | alcar, carnitine |
| 5 | B1 | b1, thiam, b 1, b-1 |
| 6 | B12 | b12, cobalamine, b-12, b 12 |
| 7 | B2 | b2, riboflavin, b 2, b-2 |
| 8 | B5 | b5, pantoth, b 5, b-5 |
| 9 | B6 | b6, pyridox, b 6, b-6 |
| 10 | D3 | vitamin d, vit d, d3, cholecalciferol, calciferol, ergocalciferol, d-3 |
| 11 | NAC | nac, acetylcysteine |
| 12 | NAD_booster | nmn, nad, nr, nicotin, niagen, niacinamid, nam |
| 13 | Q10 | q10, coenzyme, ubiquinol, ubiquinone, co q, q-10, co-q-10, ubidecarenone |
| 14 | dAKG | rejuvant |
| 15 | TMG | tmg, trimethylglycine, betaine |
| 16 | apigenin | apgenin, apigenin |
| 17 | ashwagandha | ashwagandha, ashwaganda |
| 18 | b_complex | b complex, b vitamins |
| 19 | berberine | berberine |
| 20 | beta_al | beta-alanine, beta alanine |
| 21 | biotin | biotin, vitamin b7 |
| 22 | botanicals | gingko, ginseng, echinacea, boswellia |
| 23 | calcium | ca, calcium |
| 24 | caroten | caroten |
| 25 | caroten2 | caroten, lutein, astaxa, lycope, zeaxan |
| 26 | cbd | cbd, cannabidiol |
| 27 | chlorella | chlorella |
| 28 | chrom | chrom |
| 29 | collagen | collagen |
| 30 | copper | copper |
| 31 | cranberry | cranberry, cranbery |
| 32 | creatine | creatin |
| 33 | curcumin | curcumin, tumeric, turmeric |
| 34 | dhea | dhea, dehydroepiandrosterone |
| 35 | fisetin | fisetin |
| 36 | folate | folate, folic, folinic, b9, 5-mthf, metafolin |
| 37 | ginger | ginger |
| 38 | gingko | gingk |
| 39 | ginseng | ginseng |
| 40 | glucosamine | glucosamine |
| 41 | glyc | glycine |
| 42 | glylo | glylo |
| 43 | green_tea | egcg, green tea, epigallocatechin |
| 44 | gsh | glutathione |
| 45 | hyaluron | hyaluron |
| 46 | inositol | inositol |
| 47 | iodine | iodine |
| 48 | iron | iron |
| 49 | joint | glucosamine, chondroitin, msm, methylsulfonyl, collagen |
| 50 | lipoic | alpha lipoic acid, lipoic acid |
| 51 | lith | lithium |
| 52 | lysin | lysin |
| 53 | magnesium | magnesium, mg, |
| 54 | manganese | manganese, |
| 55 | mct | mct, medium c, medium-c |
| 56 | melatonin | melatonin |
| 57 | msm | msm, methylsulfonyl |
| 58 | multi | multivitamin, multi-vitamin, multiple vitamins, vitamins, centrum |
| 59 | n3 | omega 3, fish oil, n3, epa, dha, krill oil, omega-3 |
| 60 | no_booster | arginin, citrullin, citrulin, no booster, nitric oxide |
| 61 | palmetto | palmetto |
| 62 | pchol | cholin |
| 63 | potassium | potassium, kalium |
| 64 | pqq | pyrroloquinoline |
| 65 | probiotics | probiotics, probiotic, synbiotics, probiotica |
| 66 | pser | phosphatidylserine |
| 67 | quercetin | quercetin, quercentin, quercitin |
| 68 | resveratrol | resveratrol, resveratrol, resveritrol |
| 69 | rhodiola | rhodiola |
| 70 | sam | sam, s-adenosy |
| 71 | selenium | selenium, seleno |
| 72 | shrooms | lion’s, reishi, cordyceps, hericium, mushroom |
| 73 | silymarin | silymarin, milk thistle |
| 74 | spermidine | spermidine |
| 75 | spirulina | spirulina |
| 76 | sulforaphane | sulforaphane, sulforaphane, brocco, avmacol, prostaphane, cruciferou |
| 77 | swort | st. john's wort, sankt j, st john, s wort |
| 78 | taurin | taurin |
| 79 | urolith | urolithin, urolit, mitopure |
| 80 | vit_C | vitamin c, ascorbic, ascorbat |
| 81 | vit_E | vitamin e, vit e, tocopherol, tocotri |
| 82 | vit_K | vitamin k, k2, vitamin k, mk-4, mk-7, menaquinone, phylloquinone, menatetrenone, vit k-2 |
| 83 | vitaminA | vitamin a, retinol |
| 84 | zinc | zinc, zn |

**Table S1. Supplements and their synonyms**

| Component | Criteria | Points |
| --- | --- | --- |
| General physical health | Excellent → +3 Good → +1 All other responses → 0 | 0 – 3 |
| General mental health | Excellent → +3 Good → +1 All other responses → 0 | 0 – 3 |
| Sleep quality | Yes (wakes up rested) → +1 No / blank → 0 | 0 – 1 |
| Weekly exercise frequency | ≥ 6 sessions per week → +2; 3 – 5 sessions → +1; ≤ 2 sessions → 0 | 0 – 2 |
| Smoking status | Non-smoker → +6 Smoker / former / missing → 0 | 0 – 6 |
| Dietary supplement use | Uses at least one supplement → +2 None → 0 | 0 – 2 |
| Drug use | Uses at least one prescription/OTC drug → +2 None → 0 | 0 – 2 |
| Alcohol consumption | < 11 units per week → +1; ≥ 11 units → 0 | 0 – 1 |
| Perceived stress level | Not at all → +3 Medium stress → +1 Quite a bit / Severe / blank → 0 | 0 – 3 |

**Table S2. Construction of health score**

| ID | term | Estimate | CI lower | CI upper | std.beta | p-value |
| --- | --- | --- | --- | --- | --- | --- |
| 1 | (Intercept) | 3.361 | -11.46 | 18.179 | -0.148 | 0.6565 |
| 2 | age | 0 | -0.014 | 0.015 | 0.001 | 0.96 |
| 3 | Smoke2Yes | 1.717 | 0.799 | 2.635 | 0.35 | 0.0003 |
| 4 | general_health_statusFair | 0.643 | -0.135 | 1.421 | 0.131 | 0.1054 |
| 5 | general_health_statusGood | 0.377 | -0.08 | 0.835 | 0.077 | 0.1061 |
| 6 | general_health_statusPoor | 1.81 | -0.776 | 4.395 | 0.369 | 0.17 |
| 7 | sexMale | -0.247 | -0.764 | 0.269 | -0.05 | 0.3479 |
| 8 | country2Austria | 1.451 | -0.238 | 3.141 | 0.296 | 0.0922 |
| 9 | country2Canada | 1.047 | -0.419 | 2.513 | 0.214 | 0.1615 |
| 10 | country2Great Britain/N.Ireland | 0.141 | -1.523 | 1.805 | 0.029 | 0.8678 |
| 11 | country2other/undisclosed | 0.036 | -0.979 | 1.052 | 0.007 | 0.9438 |
| 12 | country2Singapore | -3.925 | -5.976 | -1.874 | -0.801 | 0.0002 |
| 13 | country2Switzerland | -0.91 | -2.413 | 0.593 | -0.186 | 0.2351 |
| 14 | country2United States of America | 0.47 | -0.362 | 1.302 | 0.096 | 0.2684 |
| 15 | wake_up_restedYes | -0.108 | -0.559 | 0.342 | -0.022 | 0.6373 |
| 16 | has_diabetesYes | 0.398 | -0.645 | 1.442 | 0.081 | 0.4543 |
| 17 | general_mental_health_statusFair | 0.606 | -0.148 | 1.36 | 0.124 | 0.1153 |
| 18 | general_mental_health_statusGood | 0.103 | -0.351 | 0.556 | 0.021 | 0.6573 |
| 19 | general_mental_health_statusPoor | -1.774 | -3.947 | 0.4 | -0.362 | 0.1096 |
| 20 | BMI | -0.152 | -0.439 | 0.134 | -0.142 | 0.2968 |
| 21 | weight_kg | 0.047 | -0.049 | 0.143 | 0.161 | 0.3349 |
| 22 | height_m | -2.413 | -10.97 | 6.141 | -0.051 | 0.5803 |
| 23 | Weekly_exercise | 0.05 | -0.049 | 0.149 | 0.021 | 0.318 |
| 24 | stress_level.L | -0.595 | -1.577 | 0.388 | -0.121 | 0.2353 |
| 25 | stress_level.Q | -0.135 | -0.904 | 0.635 | -0.028 | 0.7311 |
| 26 | stress_level.C | -0.012 | -0.556 | 0.532 | -0.002 | 0.9652 |
| 27 | alcohol_consumption1.5 | -0.249 | -0.698 | 0.2 | -0.051 | 0.2769 |
| 28 | alcohol_consumption4 | 0.447 | -0.072 | 0.966 | 0.091 | 0.0916 |
| 29 | alcohol_consumption7.5 | -0.353 | -1.022 | 0.317 | -0.072 | 0.3016 |
| 30 | alcohol_consumption11 | -0.531 | -1.413 | 0.351 | -0.108 | 0.2381 |

**Table S3. Linear model of health-related behaviours**Multiple R-squared: 0.02819, Adjusted R-squared: 0.01757, F-statistic: 2.654 on 31 and 2836 DF, p-value: 0.000002024.

| # | Abbreviation | Drug-class description | Examples |
| --- | --- | --- | --- |
| 1 | metformin | Biguanide insulin-sensitising agent used as first-line therapy for type 2 diabetes mellitus | e.g. metformin IR, metformin XR |
| 2 | rapa | mTOR (“rapalog”) inhibitors used as immunosuppressants and experimental longevity agents | e.g. sirolimus, everolimus, temsirolimus |
| 3 | statin | HMG-CoA-reductase inhibitors that lower LDL-cholesterol and cardiovascular risk | e.g. atorvastatin, rosuvastatin, simvastatin |
| 4 | thyroid | Thyroid-hormone replacement therapies for hypothyroidism | e.g. levothyroxine (T4), liothyronine (T3), desiccated thyroid |
| 5 | pde5 | Phosphodiesterase-5 inhibitors for erectile dysfunction & pulmonary hypertension | e.g. sildenafil, tadalafil, vardenafil |
| 6 | testosterone | Testosterone-replacement & anabolic-androgenic steroid formulations | e.g. testosterone cypionate, testosterone enanthate, transdermal gel |
| 7 | bp_med | Antihypertensive medications | e.g. lisinopril (ACEi), amlodipine (CCB), hydrochlorothiazide (thiazide) |
| 8 | depression | Major oral antidepressants | e.g. sertraline (SSRI), venlafaxine (SNRI), bupropion (NDRI) |
| 9 | estrogen | Systemic estrogen or combined estrogen–progestin hormone therapy | e.g. oral conjugated estrogens, transdermal estradiol, EE/levonorgestrel COCP |
| 10 | glp1 | GLP-1 receptor agonists / dual GIP–GLP-1 agonists for diabetes & weight loss | e.g. liraglutide, semaglutide, tirzepatide |
| 11 | stimulants | CNS stimulants for ADHD & narcolepsy | e.g. mixed amphetamine salts, lisdexamfetamine, methylphenidate |
| 12 | ppi_antacid | Gastric-acid–suppressing agents | e.g. omeprazole, pantoprazole, famotidine |
| 13 | antithrombosis | Anticoagulant & antiplatelet agents preventing thrombosis | e.g. warfarin, rivaroxaban, aspirin |
| 14 | sglt2 | Sodium-glucose co-transporter-2 inhibitors for type 2 diabetes | e.g. dapagliflozin, empagliflozin, canagliflozin |
| 15 | dpp4 | Dipeptidyl-peptidase-4 inhibitors (“gliptins”) for type 2 diabetes | e.g. sitagliptin, linagliptin, saxagliptin |
| 16 | nsaid | Non-steroidal anti-inflammatory drugs (analgesic, anti-inflammatory) | e.g. ibuprofen, naproxen, celecoxib |
| 17 | antihistamine | H₁-receptor antihistamines for allergy relief | e.g. loratadine, cetirizine, diphenhydramine |
| 18 | steroid | Systemic corticosteroids with glucocorticoid activity | e.g. prednisone, methylprednisolone, dexamethasone |
| 19 | antibiotic | Common broad-spectrum oral antibiotics | e.g. amoxicillin-clavulanate, azithromycin, doxycycline |
| 20 | naltrexone | Opioid-receptor antagonist for OUD, AUD & low-dose off-label uses | e.g. naltrexone 50 mg, low-dose naltrexone |
| 21 | five_ared | 5-α-reductase inhibitors (5-ARIs) for BPH & androgenic alopecia | e.g. finasteride, dutasteride |
| 22 | benzo | Benzodiazepines – GABA-A receptor positive allosteric modulators | e.g. alprazolam, lorazepam, diazepam |

**Table S4. Medication classes included in this manuscript**Please note that some of these classes were rarely used in our cohort and were thus dropped from the final analysis.

| ID | supplement | count | frac | mean_age_delta | mean_residual | p_value | p_adj |
| --- | --- | --- | --- | --- | --- | --- | --- |
| 1 | NAD_booster | 720 | 0.169 | -2.76 | -0.20 | 0.280 | 0.785 |
| 2 | D3 | 690 | 0.162 | -3.09 | -0.38 | 0.038 | 0.497 |
| 3 | n3 | 522 | 0.123 | -2.37 | -0.11 | 0.575 | 0.940 |
| 4 | magnesium | 479 | 0.112 | -1.85 | 0.54 | 0.016 | 0.418 |
| 5 | multi | 473 | 0.111 | -2.61 | -0.19 | 0.397 | 0.870 |
| 6 | resveratrol | 260 | 0.061 | -2.94 | -0.45 | 0.181 | 0.717 |
| 7 | vit_K | 245 | 0.058 | -2.63 | 0.07 | 0.808 | 0.940 |
| 8 | zinc | 226 | 0.053 | -2.87 | -0.13 | 0.705 | 0.940 |
| 9 | AKG_any | 217 | 0.051 | -4.87 | -1.21 | 0.001 | 0.037 |
| 10 | Q10 | 213 | 0.05 | -3.53 | -0.16 | 0.614 | 0.940 |
| 11 | joint | 210 | 0.049 | -2.46 | -0.42 | 0.244 | 0.775 |
| 12 | curcumin | 193 | 0.045 | -3.38 | 0.01 | 0.977 | 0.982 |
| 13 | B1 | 188 | 0.044 | -3.20 | -0.42 | 0.276 | 0.785 |
| 14 | B12 | 177 | 0.042 | -3.16 | -0.49 | 0.232 | 0.775 |
| 15 | collagen | 150 | 0.035 | -2.10 | -0.74 | 0.094 | 0.597 |
| 16 | probiotics | 144 | 0.034 | -2.75 | -0.80 | 0.085 | 0.597 |
| 17 | dAKG | 143 | 0.034 | -5.74 | -1.81 | 0.000 | 0.013 |
| 18 | calcium | 137 | 0.032 | -4.20 | -0.38 | 0.406 | 0.870 |
| 19 | NAC | 136 | 0.032 | -2.69 | 0.16 | 0.688 | 0.940 |
| 20 | quercetin | 128 | 0.03 | -2.42 | 0.26 | 0.564 | 0.940 |
| 21 | vit_C | 118 | 0.028 | -3.02 | -0.49 | 0.255 | 0.775 |
| 22 | berberine | 94 | 0.022 | -2.95 | 0.18 | 0.723 | 0.940 |
| 23 | b_complex | 89 | 0.021 | -2.38 | 0.77 | 0.149 | 0.654 |
| 24 | AKG_not_delayed | 81 | 0.019 | -3.13 | -0.22 | 0.666 | 0.940 |
| 25 | ALA | 81 | 0.019 | -2.62 | 0.38 | 0.470 | 0.919 |
| 26 | sam | 81 | 0.019 | -2.90 | 0.32 | 0.536 | 0.940 |
| 27 | TMG | 83 | 0.019 | -2.08 | 0.85 | 0.112 | 0.597 |
| 28 | creatine | 74 | 0.017 | -1.32 | -0.33 | 0.477 | 0.919 |
| 29 | fisetin | 63 | 0.015 | -1.89 | 0.34 | 0.595 | 0.940 |
| 30 | glucosamine | 66 | 0.015 | -3.02 | 0.14 | 0.822 | 0.940 |
| 31 | iron | 64 | 0.015 | -1.69 | -0.48 | 0.440 | 0.892 |
| 32 | dhea | 59 | 0.014 | -1.90 | 1.16 | 0.061 | 0.597 |
| 33 | melatonin | 59 | 0.014 | -3.36 | -1.39 | 0.054 | 0.597 |
| 34 | caroten2 | 57 | 0.013 | -4.18 | -0.23 | 0.751 | 0.940 |
| 35 | biotin | 51 | 0.012 | -2.84 | -1.01 | 0.246 | 0.775 |
| 36 | folate | 48 | 0.011 | -2.63 | 0.02 | 0.968 | 0.982 |
| 37 | ashwagandha | 42 | 0.01 | -1.83 | 0.11 | 0.878 | 0.940 |
| 38 | glyc | 42 | 0.01 | -3.02 | -0.11 | 0.875 | 0.940 |
| 39 | spermidine | 38 | 0.009 | -2.53 | -0.39 | 0.631 | 0.940 |
| 40 | potassium | 32 | 0.008 | -1.44 | 1.44 | 0.312 | 0.785 |
| 41 | selenium | 34 | 0.008 | -5.37 | -1.09 | 0.181 | 0.717 |
| 42 | taurin | 31 | 0.007 | -3.02 | 0.22 | 0.792 | 0.940 |
|  |  |  |  |  |  |  |  |

**Table S5. Commonly consumed supplements**The table shows the number (count) and the fraction (frac) of participants that consume a certain supplement or supplement class. Only data for supplements consumed by more than 30 participants shown.

| **variable** | **level** | **Non-user** | **User** | **p** |
| --- | --- | --- | --- | --- |
| N |  | 2498 | 1762 |  |
| age |  | 52.99 (13.10) | 54.20 (13.17) | 0.003 |
| health status | Poor | 32 (1.6) | 5 (0.3) | <0.001 |
|  | Fair | 266 (13.4) | 147 (8.8) |  |
|  | Good | 1000 (50.5) | 838 (50.1) |  |
|  | Excellent | 684 (34.5) | 683 (40.8) |  |
| smoking | No | 2094 (95.7) | 1689 (96.9) | 0.061 |
|  | Yes | 94 (4.3) | 54 (3.1) |  |
| sex | Female | 1026 (41.2) | 736 (41.8) | 0.744 |
|  | Male | 1463 (58.8) | 1026 (58.2) |  |

**Table S6. Baseline characteristics of supplement users and non-users**

| **variable** | **level** | **0** | **1** | **p** |
| --- | --- | --- | --- | --- |
| N |  | 4117 | 143 |  |
| age |  | 53.26 (13.11) | 60.17 (12.32) | <0.001 |
| health status | Poor | 37 (1.1) | 0 (0.0) | 0.225 |
|  | Fair | 402 (11.4) | 11 (8.0) |  |
|  | Good | 1771 (50.4) | 67 (48.6) |  |
|  | Excellent | 1307 (37.2) | 60 (43.5) |  |
| smoking | No | 3642 (96.1) | 141 (98.6) | 0.197 |
|  | Yes | 146 (3.9) | 2 (1.4) |  |
| sex | Female | 1706 (41.5) | 56 (39.2) | 0.632 |
|  | Male | 2402 (58.5) | 87 (60.8) |  |

**Table S7. Baseline characteristics of dAKG users and non-users**

| term | Estimate | CI lower | CI upper | std.beta | p-value |
| --- | --- | --- | --- | --- | --- |
| (Intercept) | -1.242 | -2.814 | 0.33 | -0.165 | 0.1214 |
| Smoke2Yes | 0.928 | -0.046 | 1.902 | 0.216 | 0.0618 |
| age | 0.01 | -0.004 | 0.024 | 0.031 | 0.1544 |
| general_health_statusFair | 0.946 | 0.179 | 1.712 | 0.22 | 0.0156 |
| general_health_statusGood | 0.49 | 0.066 | 0.913 | 0.114 | 0.0234 |
| sexMale | -0.007 | -0.371 | 0.358 | -0.002 | 0.9704 |
| brand_name3Rejuvant | 0.2 | -0.325 | 0.725 | 0.047 | 0.4548 |
| brand_name3TruMe Labs | -0.345 | -0.751 | 0.061 | -0.08 | 0.0959 |
| country2Canada | -0.252 | -2.244 | 1.741 | -0.059 | 0.8043 |
| country2other/undisclosed | -0.155 | -1.109 | 0.798 | -0.036 | 0.7495 |
| country2Switzerland | -1.081 | -2.527 | 0.365 | -0.251 | 0.1428 |
| country2United States of America | 0.51 | -0.288 | 1.308 | 0.119 | 0.2105 |
| wake_up_restedYes | -0.034 | -0.461 | 0.393 | -0.008 | 0.8754 |
| general_mental_health_statusFair | 0.33 | -0.41 | 1.07 | 0.077 | 0.3818 |
| general_mental_health_statusGood | 0.23 | -0.193 | 0.652 | 0.053 | 0.2864 |
| BMI | -0.009 | -0.052 | 0.034 | -0.009 | 0.6815 |
| Weekly_exercise | 0.045 | -0.049 | 0.14 | 0.021 | 0.3498 |
| stress_level.L | -0.13 | -0.668 | 0.409 | -0.03 | 0.6367 |
| stress_level.Q | 0.078 | -0.262 | 0.418 | 0.018 | 0.6535 |
| alcohol_consumption1.5 | -0.132 | -0.551 | 0.288 | -0.031 | 0.5385 |
| alcohol_consumption4 | 0.311 | -0.172 | 0.793 | 0.072 | 0.207 |
| alcohol_consumption7.5 | -0.413 | -1.041 | 0.216 | -0.096 | 0.198 |
| alcohol_consumption11 | 0.301 | -0.582 | 1.185 | 0.07 | 0.5038 |
| dAKG | -1.721 | -2.804 | -0.638 | -0.065 | 0.0019 |

**Table S8. Linear model for dAKG effects**Multiple R-squared: 0.0194, Adjusted R-squared: 0.0103, F-statistic: 2.12 on 23 and 2462 DF, p-value: 0.00144. We quantified potential outliers using Cook’s distance and leverage values from the fitted model, and defined potentially influential observations as those with Cook’s distance greater than 4/n or leverage greater than twice the mean leverage. Observations meeting either of these criteria were flagged as influential for downstream analyses. A total of 355 outlier rows were dropped to improve kurtosis and skewness of the data. The model with and without outliers produced similar results.

| term | estimate | SE | t | p | CI_low | CI_high |
| --- | --- | --- | --- | --- | --- | --- |
| (Intercept) | 1.245 | 0.260 | 4.778 | 0.000 | 0.734 | 1.756 |
| timepointlast | -1.587 | 0.263 | -6.038 | 0.000 | -2.103 | -1.071 |
| curcumin | 0.030 | 0.581 | 0.052 | 0.958 | -1.109 | 1.170 |
| Q10 | -0.018 | 0.525 | -0.034 | 0.973 | -1.047 | 1.012 |
| calcium | 0.083 | 0.702 | 0.119 | 0.905 | -1.294 | 1.461 |
| joint | 0.042 | 0.654 | 0.064 | 0.949 | -1.241 | 1.324 |
| dAKG_subscription | 0.062 | 0.289 | 0.215 | 0.830 | -0.505 | 0.630 |
| dAKG | 0.154 | 0.722 | 0.213 | 0.831 | -1.263 | 1.571 |
| age_c | -0.010 | 0.008 | -1.212 | 0.226 | -0.026 | 0.006 |
| sexFemale | -0.184 | 0.202 | -0.910 | 0.363 | -0.581 | 0.213 |
| sexother/no_data | -0.905 | 2.533 | -0.357 | 0.721 | -5.875 | 4.064 |
| Smoke2Yes | 0.540 | 0.538 | 1.005 | 0.315 | -0.514 | 1.595 |
| general_health_statusExcellent | -0.446 | 0.211 | -2.109 | 0.035 | -0.860 | -0.031 |
| general_health_statusFair | -0.738 | 0.361 | -2.046 | 0.041 | -1.446 | -0.030 |
| general_health_statusPoor | -0.513 | 1.044 | -0.491 | 0.623 | -2.561 | 1.535 |
| general_health_statusother/no_da | -0.050 | 0.412 | -0.121 | 0.903 | -0.859 | 0.758 |
| date_diff_val | -0.001 | 0.000 | -3.397 | 0.001 | -0.002 | 0.000 |
| baseline_bioage | 6.258 | 0.138 | 45.223 | 0.000 | 5.986 | 6.529 |
| timepointlast:curcumin | -0.567 | 0.819 | -0.692 | 0.489 | -2.174 | 1.041 |
| timepointlast:Q10 | -0.529 | 0.738 | -0.717 | 0.474 | -1.976 | 0.918 |
| timepointlast:calcium | 0.675 | 0.991 | 0.681 | 0.496 | -1.268 | 2.618 |
| timepointlast:joint | 0.624 | 0.922 | 0.677 | 0.499 | -1.185 | 2.433 |
| timepointlast:dAKG_subscripti | -0.460 | 0.397 | -1.157 | 0.247 | -1.239 | 0.320 |
| timepointlast:dAKG | -0.311 | 1.015 | -0.307 | 0.759 | -2.302 | 1.680 |
| timepointlast:baseline_bioage | -4.187 | 0.195 | -21.516 | 0.000 | -4.569 | -3.805 |

**Table S9. Mixed linear model for dAKG effects**Formula: marker ~ timepoint * (curcumin + Q10 + calcium + joint + dAKG_subscription + dAKG + baseline_bioage) + age_c + sex + smoking + general_health_status + date_diff_val+ (1 | participant_id).
